# Identification of Dihydropyrazolo[1,5-*a*]pyrazin-4(5*H*)-ones as Cyclic Products of β-Amidomethyl Vinyl Sulfone Alphavirus Cysteine Protease Inhibitors

**DOI:** 10.3390/ph17070836

**Published:** 2024-06-26

**Authors:** Anirban Ghoshal, Álvaro F. Magalhães, Kesatebrhan Haile Asressu, Mohammad Anwar Hossain, Matthew H. Todd, Timothy M. Willson

**Affiliations:** 1 Structural Genomics Consortium, UNC Eshelman School of Pharmacy, University of North Carolina at Chapel Hill, Chapel Hill, NC 27599, USA; 2 Structural Genomics Consortium, Department of Pharmaceutical and Biological Chemistry, School of Pharmacy, University College London, London WC1N 1AX, UK

**Keywords:** cysteine protease, covalent inhibitor, vinyl sulfone, antiviral, prodrug

## Abstract

Optimized syntheses of (*E*)-5-(2-ethoxyphenyl)-*N*-(3-(methylsulfonyl)allyl)-1*H*-pyrazole-3-carboxamide (RA-0002034, **1**), a promising antiviral covalent cysteine protease inhibitor lead, were developed. The syntheses avoid the contamination of **1** with the inactive cyclic dihydropyrazolo[1,5-*a*]pyrazin-4(5*H*)-one **2**, which is formed by the intramolecular aza-Michael reaction of the vinyl sulfone warhead under basic conditions and slowly at pH 7.4 in phosphate buffer. The pure cysteine protease inhibitor **1** could be synthesized using either modified amide coupling conditions or through the introduction of a MOM-protecting group and was stable as a TFA or HCl salt. Although acyclic **1** demonstrated poor pharmacokinetics with high in vivo clearance in mice, inactive cyclic **2** showed improved plasma exposure. The potential use of cyclic dihydropyrazolo[1,5-*a*]pyrazin-4(5*H*)-ones as prodrugs for the acyclic β-amidomethyl vinyl sulfone warhead was demonstrated by GSH capture experiments with an analog of **2**.

## 1. Introduction

Alphaviruses, a group of widespread, enveloped, single-stranded positive sense RNA viruses, are transmitted by *Aedes aegypti* and *Aedes albopictus* mosquitoes, posing a significant threat to public health [1]. These viruses are divided into two categories based on their geographical emergence: Old World alphaviruses, including Chikungunya virus (CHIKV), Ross River virus (RRV), and O’nyong-nyong virus (ONNV) that typically present with rash, fever, and prolonged arthralgia that can persist for months post-infection [2]; and New World alphaviruses, including Venezuelan (VEEV), Western (WEEV), and Eastern (EEEV) Equine Encephalitis viruses and Mayaro virus (MAYV), that often result in encephalitis-like neurological symptoms, accompanied by fever, headache, and nausea, which can be fatal, with 30–50% of EEEV cases resulting in mortality [3]. Despite the severity of these diseases, there are currently no FDA-approved drugs for any alphavirus-caused disease, highlighting the urgent need for the development of alphavirus therapeutics.

**Figure 1:**
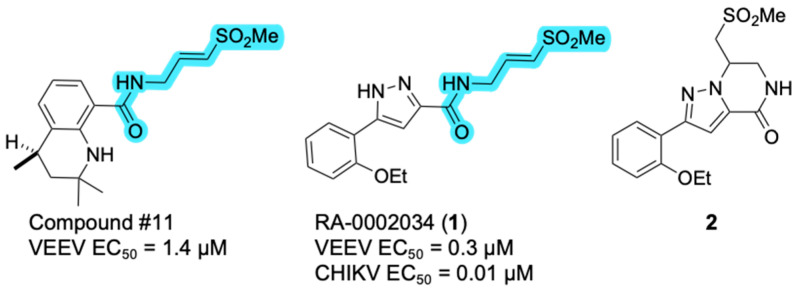
Alphavirus nsP2 protease inhibitors. The vinyl sulfone covalent warhead in compound #11 (ref. [5]) and RA-0002034 (**1**) is highlighted in blue. Potencies for inhibition of alphavirus replication are indicated.

The largest non-structural protein in the alphavirus genome, nsP2, is essential for viral replication [4]. nsP2 contains a C-terminal cysteine protease that uses a catalytic dyad of cysteine and histidine residues to catalyze substrate cleavage. Two covalent inhibitors of alphavirus nsP2 protease that contain a common β-amidomethyl vinyl sulfone warhead have been recently disclosed (Figure 1). Compound #11 was reported as a micromolar inhibitor of VEEV nsP2 protease activity with antiviral activity [5]. Likewise, we reported the discovery of RA-0002034 (**1**) as a potent covalent inhibitor of the CHIKV nsP2 protease with IC_50_ = 60 nM [6]. Vinyl sulfone **1** inhibited VEEV and CHIKV replication with EC_50_ = 0.3 and 0.01 µM, respectively, and decreased viral titer across a wide range of New and Old World alphaviruses [6]. Notably, the 5-arylpyrazole in **1** conferred an increase in potency for nsP2 protease inhibition compared to the 1,2-dihydroquinoline in Compound #11, demonstrating the importance of the heterocyclic amide substituent in molecular recognition by the viral protease.

Vinyl sulfones have broad utility as covalent inhibitors of cysteine proteases beyond viral nsP2 [7]. However, the cysteine reactivity of these warheads must be balanced with concerns of toxicity due to off-target activity or poor pharmacokinetics due to systemic GSH reactivity [8]. During the resynthesis of **1**, we observed the formation of a cyclic byproduct **2** that effectively masked the vinyl sulfone warhead, rendering it inactive as an nsP2 protease inhibitor. In this report, we document methods to synthesize pyrazole-substituted β-amidomethyl vinyl sulfones, such as **1**, devoid of contamination from cyclic dihydropyrazolo[1,5-*a*]pyrazin-4(5*H*)-ones. We also explored the reversibility of the cyclization reaction as a potential prodrug strategy for the cysteine-reactive acyclic β-amidomethyl vinyl sulfone.

## 2. Results and Discussion

### 2.1. Identification of a Cyclic Product of Pyrazole-Substituted β-Amidomethyl Vinyl Sulfone ***1***

**Scheme 1:**
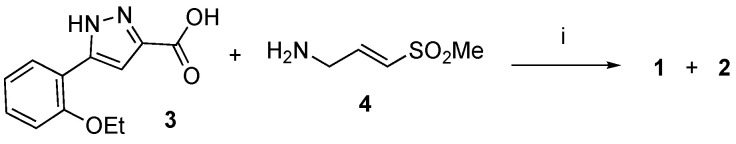
(i) HBTU, HOBt, DIPEA, DMF, 25 °C, 16 h.

Synthesis of an nsP2 protease inhibitor (*E*)-5-(2-ethoxyphenyl)-*N*-(3-(methylsulfonyl)allyl)-1*H*-pyrazole-3-carboxamide (**1**) from 3-carboxypyrazole (**3**) was attempted by amide coupling with (*E*)-3-(methylsulfonyl)prop-2-en-1-amine **4** using hexafluorophosphate benzotriazole tetramethyl uronium (HBTU), 1-hydroxybenzotriazole (HOBt), and diisopropylethylamine (DIPEA) in dimethylformamide (DMF). ^1^H NMR analysis of the isolated product indicated an approx. 1:1 mixture of the expected product **1** together with a byproduct **2** that was inseparable by thin layer and column chromatography (Scheme 1).

**Figure 2:**
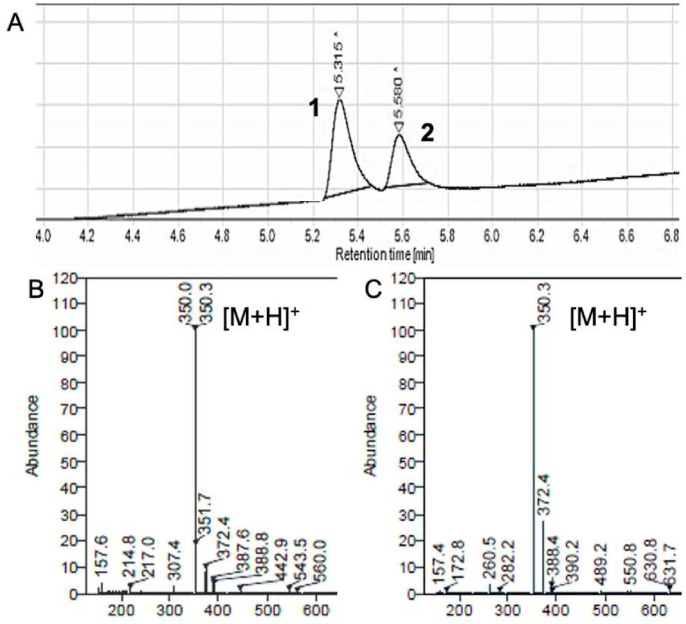
LCMS analysis of **1** and byproduct **2**. (**A**) Analytical UPLC separation of **1** and **2** using a reverse-phase C18 2.7 μm column (Agilent (Santa Clara, CA, USA)). (**B**) Positive ion mass spectrum of **1**, *m*/*z*: [M + H]^+^ = 350. (**C**) Positive ion mass spectrum of **2**, *m*/*z*: [M + H]^+^ = 350.

LCMS analysis using an Ultrahigh Performance Liquid Chromatography (UPLC) reverse phase C18 column with an extended run time achieved baseline separations of **1** and **2** (Figure 2A). The mass spectra of the respective peaks showed predicted molecular weights of 349 Da for both compounds (Figure 2B,C), indicating that they were likely to be constitutional isomers. Preparative HPLC separation using a reverse phase Luna 5 μm phenyl-hexyl column (Phenomenex, Torrance, CA, USA) provided high purity (>99%) samples of **1** and **2** as their TFA salts. CHIKV nsP2 protease inhibition was found to reside exclusively in **1** (IC_50_ = 60 nM), with the byproduct **2** devoid of activity in the enzyme assay at 200 µM (Table S1).

**Figure 3:**
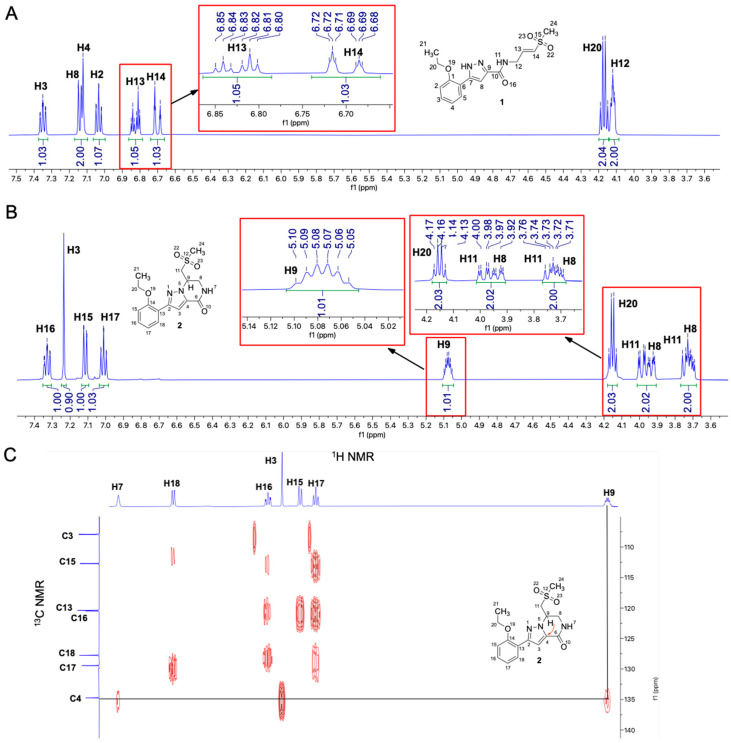
(**A**) 500 MHz ^1^H NMR spectra in DMSO-d_6_. **1** has diagnostic olefin signals H13 and H14. (**B**) **2** has diagnostic methine signal H9 and methylene signals for H8 and H11. (**C**) ^1^H-^13^C HMBC spectrum of **2**, indicating the three-bond correlation between atoms H9 and C4.

Using a combination of ^1^H and ^13^C NMR spectroscopy, the chemical structure of **2** was determined to be a dihydropyrazolo[1,5-*a*]pyrazin-4(5*H*)-one (Figure 1) arising from intramolecular cyclization of **1**. ^1^H NMR analysis of **1** showed characteristic olefinic protons corresponding to the (*E*)-vinyl sulfone at *δ* 6.69 ppm (dt, *J* = 15.3, 1.8 Hz, H14) and *δ* 6.82 ppm (dt, *J* = 15.3, 4.4 Hz, H13) (Figure 3A). These olefin resonances were absent in **2** (Figure 3B) and were replaced by a multiplet at *δ* 5.07 ppm (m, *J* = 8.9, 4.5 Hz, H9) and signals for two protons at *δ* 3.76–3.72 ppm (m, H11) and *δ* 3.98 ppm (dd, *J* = 14.4, 3.9 Hz, H11). Other resonances consistent with the cyclic structure of **2** were the non-equivalent methylene protons at *δ* 3.70 ppm (td, *J* = 6.3, 3.5 Hz, H8) and *δ* 3.93 ppm (ddd, *J* = 13.3, 4.4, 2.3 Hz, H8) (Figure 3B), which appeared as a single multiplet at *δ* 4.11 ppm in acyclic **1** (Figure 3A, H12). Additional evidence for the cyclic structure of **2** was provided by a ^1^H-^13^C heteronuclear multiple bond correlation (HMBC) experiment (Figure 3C) that indicated a three-bond correlation between atoms H9 and C4 in **2** that was absent in the acyclic **1**.

**Scheme 2:**
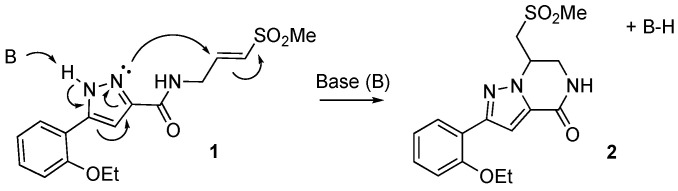
Proposed mechanism of cyclization of **1** to **2** under basic conditions.

Formation of **2** is proposed to occur under the basic conditions of the amide coupling by a formal aza-Michael conjugate addition [9,10] of the pyrazole N2 into the β-carbon of the vinyl sulfone (Scheme 2). The resulting dihydropyrazolo[1,5-*a*]pyrazin-4(5*H*)-one **2**, which masks the reactive vinyl sulfone warhead within its cyclic structure, was stable under normal laboratory conditions with no propensity to revert to the acyclic **1** upon storage as a solid or as a 10 mM DMSO stock solution. Unsurprisingly, **2** was inactive as an nsP2 protease inhibitor at concentrations up to 200 μM (Table S1).

### 2.2. Stability of ***1*** in Neutral Phosphate Buffer

**Figure 4:**
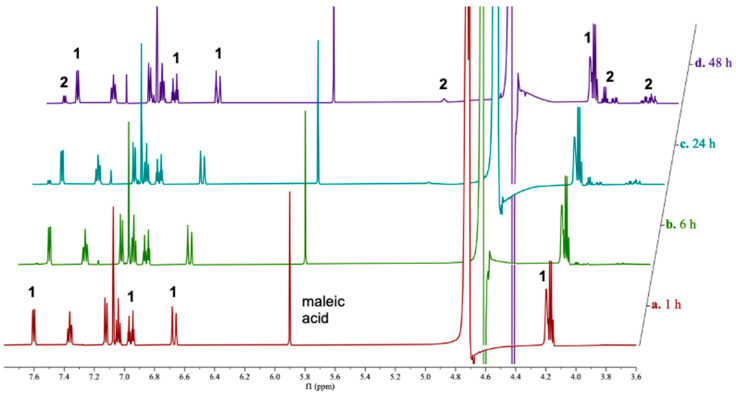
Cyclization of **1** in aqueous media. ^1^H NMR (600 MHz, H_2_O/D_2_O 9:1, noesygppr1d, 3.6–7.8 ppm) of vinyl sulfone (**1**, 2 mM) in phosphate buffer (pH 7.4, 200 mM), with maleic acid (1 mM) as an internal standard after: **a**. 1 h; **b**. 6 h; **c**. 24 h; **d**. 48 h. Diagnostic signals for **1** and **2** are indicated.

To assess if cyclization of **1** into **2** could occur during the assessment of cellular antiviral activity, the stability of **1** in neutral pH phosphate buffer was investigated. To achieve this, a stock solution of **1** was prepared in DMSO-*d_6_* and diluted into pH 7.4 phosphate buffer (with 10% D_2_O) to a final concentration of 2 mM in the presence of maleic acid as an internal standard. The solution was analyzed by ^1^H NMR spectroscopy over 48 h at room temperature (Figure 4). At t = 1 h, vinyl sulfone **1** was present in solution at the expected 2 mM concentration. After 24 h, a reduction in the abundance of **1** by 10% and the appearance of approximately 9% of its cyclic isomer **2** were observed. After 48 h, 81% of **1** remained, with approximately 19% of **2** present in the phosphate buffer.

These results demonstrated that partial cyclization of **1** into **2** might occur under standard cell culture conditions. Whether this decrease in the effective concentration of **1** would influence its efficacy as an antiviral nsP2 inhibitor would depend on the frequency of dosing and the time course of the bioassay.

### 2.3. Optimized Synthesis of β-Amidomethyl Vinyl Sulfone ***1***

Alternative amide coupling conditions [11] were explored for the synthesis of acyclic β-amidomethyl vinyl sulfone **1** that would avoid the formation of cyclic byproduct **2** and the subsequent preparative HPLC separation (Table 1). The original conditions using HBTU as the coupling agent and DIPEA as the base in DMF yielded a 60:40 mixture of **1** and **2** by analytical UPLC analysis (entry 1, Table 1). Switching the coupling agent to hexafluorophosphate azabenzotriazole tetramethyl uronium (HATU) or benzotriazol-1-yl-oxytripyrrolidino-phosphonium hexafluorophosphate (PyBOP) resulted in a similar ratio of **1** and **2** (entries 2 and 3). Use of *n*-propanephosphonic acid anhydride (T3P) as the coupling agent with triethylamine (TEA) as the base also yielded a 60:40 ratio of **1** and **2**, as did *N*,*N*′-diisopropylcarbodiimide (DIC) with 4-dimethylaminopyridine (DMAP) (entries 4 and 5). Each of these amide coupling reactions (entries 1–5) occurred in the presence of strongly basic amines with pK_a_ 9.7–10.9. Switching to 1-ethyl-3-(3-dimethylaminopropyl)carbodiimide (EDC) in combination with hydroxybenzotriazole (HOBt), where amide coupling occurs without the addition of an amine base, resulted in an improved 70:30 ratio of **1** and **2** (entry 6). This result prompted us to trial the amide synthesis with the original benzotriazole tetramethyl uronium coupling agent but as a tetrafluoroborate salt (TBTU) in pyridine as a solvent. Under these less basic conditions (entry 7), exclusive formation of **1** was observed by UPLC analysis.

**Scheme 3:**
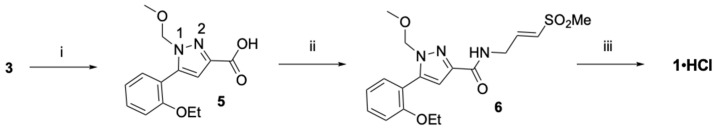
(i) MOMCl, K_2_CO_3_, DMSO (ii) **5**, TBTU, pyridine (iii) HCl, dioxane.

An alternate synthesis of β-amidomethyl vinyl sulfone **1** was also developed to avoid the liability of cyclization during the amide bond formation (Scheme 3). MOM-protection of pyrazole **3** occurred exclusively at the N1 position as expected [12,13] and was confirmed by ^1^H-^13^C HMBC NMR analysis. The coupling of MOM-protected pyrazole **5** using the TBTU-pyridine protocol gave exclusively amide **6** with no byproducts resulting from cyclization. Acid-mediated cleavage of the MOM protecting group yielded pure **1** as an HCl salt without the need for chromatography. The HCl salt of **1** was stable upon storage as a solid or as a 10 mM DMSO stock solution and was routinely checked for purity prior to bioassay. However, researchers are cautioned that commercial samples of **1** or other heterocyclic β-amidomethyl vinyl sulfones (ChemSpace, Enamine) may contain undetermined quantities of the respective dihydropyrazolo[1,5-*a*]pyrazin-4(5*H*)-ones unless careful quality control by ^1^H NMR and UPLC analysis has been performed.

### 2.4. Optimized Synthesis of Dihydropyrazolo[1,5-a]pyrazin-4(5H)-one ***2***

Conditions for controlled cyclization of the HCl salt of β-amidomethyl vinyl sulfone **1** to dihydropyrazolo[1,5-*a*]pyrazin-4(5*H*)-one **2** by intramolecular aza-Michael reaction were explored (Table 2). Reactions were performed at room temperature for 2 h in the presence of different bases, with the conversion to **2** monitored by UPLC. K_2_CO_3_ in ethanol gave efficient cyclization (entry 1). However, no cyclization occurred with K_2_CO_3_ in water due to the limited aqueous solubility of **1** (entry 2). Na_2_CO_3_ in an aqueous dioxane mixture resulted in clean cyclization to **2** (entry 3). The less basic NaHCO_3_ in methanol or water was not as effective as K_2_CO_3_ (entries 4 and 5) within the 2 h reaction time. The amine base TEA in methanol was also less effective (entry 6). The stronger amine bases, DIPEA or DBU, showed faster conversion to **2** but were still not as rapid as the inorganic bases (entries 7 and 8). Na_2_CO_3_ in an aqueous dioxane (entry 3) was chosen as the optimal cyclization conditions for the synthesis of **2** since it was fast, resulted in a simple work-up, and avoided the need for chromatography.

### 2.5. Pharmacokinetic Properties of ***1*** and ***2***

**Figure 5:**
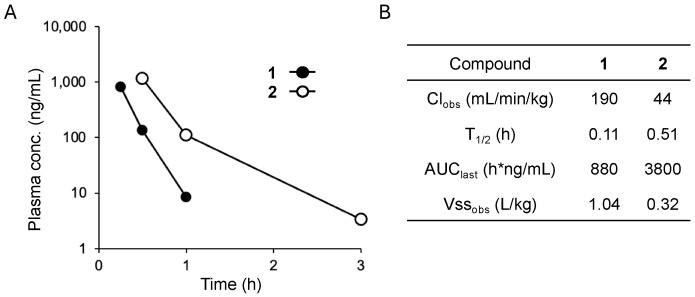
Pharmacokinetics of 10 mg/kg i.v. doses of **1** or **2** in mice. (**A**) Pooled plasma concentrations. In vivo formation of **2** from **1** was not detected. (**B**) Calculated pharmacokinetic parameters.

Although vinyl sulfones have been used as covalent warheads for inhibition of a wide range of cysteine proteases [7,8], there are relatively few reports of their use in vivo [14]. To explore the potential of **1** as a drug lead for the treatment or prevention of alphavirus infections, pharmacokinetic experiments were performed in mice following a 10 mg/kg i.v. dose (Figure 5).

Unfortunately, β-amidomethyl vinyl sulfone **1** had very rapid clearance in mice, with plasma levels falling below the limit of MS detection after 1 h and a half-life of only ~10 min. As a control, the pharmacokinetics of the cyclic dihydropyrazolo[1,5-*a*]pyrazin-4(5*H*)-one **2** were determined (Figure 2). In comparison to **1**, the cyclic pyrazole **2** showed reduced plasma clearance with an i.v. half-life of ~30 min and a 4-fold greater plasma exposure. Reanalysis of the plasma samples from the dosing of **1** showed no detectable levels of **2**, demonstrating that the rapid clearance of **1** in vivo was not due to extensive cyclization to **2**. Instead, the rapid clearance of **1** was likely due to its β-amidomethyl vinyl warhead, which appeared to be a liability for in vivo exposure in mice. Despite the fact that extensive interconversion between **1** and **2** was not observed in vivo, the improved pharmacokinetics of the cyclic dihydropyrazolo[1,5-*a*]pyrazin-4(5*H*)-one raised the question of whether it might still function as a prodrug if low levels of the reactive vinyl sulfone could be formed in the presence of the viral enzyme.

### 2.6. Reversibility of the Aza-Michael Reaction

**Scheme 4:**
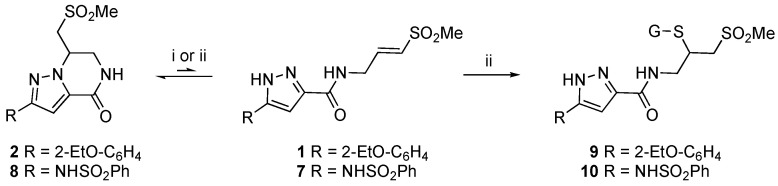
(i) NaHCO_3_ (1.0 eq.), MeOH, 25 °C. (ii) GSH (100×), phosphate buffer pH 7.4, 30 °C.

Cyclization of **1** to **2** was favored under basic conditions but occurred only slowly at physiological pH. We were eager to determine if the cyclization was reversible and under what conditions the dihydropyrazolo[1,5-*a*]pyrazin-4(5*H*)-one **2** could revert to the acyclic β-amidomethyl vinyl sulfone **1** by a retro-Michael reaction (Scheme 4).

The stability of the dihydropyrazolo[1,5-*a*]pyrazin-4(5*H*)-one **2** under standard laboratory conditions suggested that equilibrium with the acyclic β-amidomethyl vinyl sulfone **1** lay almost entirely toward the cyclic form. For example, **1** was below the limits of UPLC detection in 10 mM DMSO stock solutions of **2** even after prolonged storage over several months. Since low levels of the electrophilic β-amidomethyl vinyl sulfone could theoretically still be present, we decided to test whether **1** could be captured using glutathione (GSH). Incubation of **1** with a 100-fold excess of GSH in phosphate buffer at 30 °C led to complete conversion to GSH adduct **9** within 8 h, as monitored by LCMS (Table 3 and Figure S1). In contrast, incubation of dihydropyrazolo[1,5-*a*]pyrazin-4(5*H*)-one **2** with excess GSH did not result in the formation of the GSH adduct **9** even after 24 h. The GSH capture data demonstrated that no detectable level of the acyclic β-amidomethyl vinyl sulfone **1** was present in phosphate buffer, making it extremely unlikely that dihydropyrazolo[1,5-*a*]pyrazin-4(5*H*)-one **2**, despite its improved pharmacokinetic properties, would be useful as a prodrug for **1**.

Notably, during structure-activity studies of the pyrazole β-amidomethyl vinyl sulfones [15], a phenylsulfonamide-5-substituted pyrazole analog **7** was synthesized that appeared to be less prone to intramolecular cyclization. Under cyclization conditions of NaHCO_3_ (1.0 eq.) in MeOH, **1** was 100% converted to **2** in 36 h, but under the same conditions, only ~50% of **7** cyclized to **8**. These results suggested that the structure of the pyrazole could influence the rate of aza-Michael cyclization and possibly the propensity for the reverse reaction. To explore this hypothesis, an additional series of GSH capture experiments was performed. Incubation of β-amidomethyl vinyl sulfone **7** with a 100-fold excess of GSH in phosphate buffer at 30 °C led to the formation of its corresponding GSH adduct **10**, although conversion was slower than was seen with **1** and required 24 h to complete. More importantly, incubation of the corresponding dihydropyrazolo[1,5-*a*]pyrazin-4(5*H*)-one **8** with GSH resulted in the formation of 3% of the GSH adduct **10** after 8 h and 6% after 24 h (Figure S1). These GSH capture experiments demonstrate that the cyclic dihydropyrazolo[1,5-*a*]pyrazin-4(5*H*)-one **8** exists in equilibrium with the acyclic β-amidomethyl vinyl sulfone **7** in phosphate buffer. Unfortunately, the vinyl sulfone **7** was not sufficiently active as an nsP2 protease inhibitor (IC_50_~4 μM [15]) to allow us to test whether its formation from the cyclic form **8** would result in antiviral activity. However, our demonstration of the reversibility of the aza-Michael reaction at physiological pH adds credence to the potential use of dihydropyrazolo[1,5-*a*]pyrazin-4(5*H*)-ones as prodrugs for their corresponding cysteine reactive β-amidomethyl vinyl sulfones.

In conclusion, (*E*)-5-(2-ethoxyphenyl)-*N*-(3-(methylsulfonyl)allyl)-1*H*-pyrazole-3-carboxamide (RA-0002034, **1**) is a covalent inhibitor of nsP2 cysteine proteases with potent antiviral activity against New and Old World alphaviruses. Although **1** was prone to intramolecular cyclization to a cyclic dihydropyrazolo[1,5-*a*]pyrazin-4(5*H*)-one **2** under basic conditions, two modified procedures were developed for the synthesis of the pure acyclic β-amidomethyl vinyl sulfones as their TFA or HCl salts that can be employed in analog development for structure-activity studies of nsP2 protease inhibitors. The primary liability of **1** as an anti-alphavirus drug lead was its very high clearance in mice, which prompted us to explore the use of the inactive dihydropyrazolo[1,5-*a*]pyrazin-4(5*H*)-one **2** as a potential prodrug. Although cyclic **2** showed no evidence for a retro aza-Michael reaction to give **1**, the phenylsulfonamide-5-substituted pyrazole analog **8** formed detectable levels of its acyclic β-amidomethyl vinyl sulfone **7**, as evidenced by capture with GSH. These results demonstrate that the dihydropyrazolo[1,5-*a*]pyrazin-4(5*H*)-one chemotype can function as a masked form of the cysteine-reactive β-amidomethyl vinyl sulfone. Synthesis of dihydropyrazolo[1,5-*a*]pyrazin-4(5*H*)-one analogs with substituents that further favor equilibrium with their acyclic vinyl sulfones may provide a new prodrug strategy for covalent inhibition of viral nsP2 cysteine proteases.

## 3. Materials and Methods

### 3.1. General Methods

All reactions were performed in oven-dried glassware under an atmosphere of dry N_2_ unless otherwise stated. All reagents and solvents used were purchased from commercial sources and were used without further purification. No unexpected safety hazards were encountered during chemical synthesis. Analytical thin layer chromatography (TLC) was performed on pre-coated silica gel plates, 200 μm, with an F_254_ indicator. TLC plates were visualized by fluorescence quenching under UV light or by staining with iodine and KMnO_4_. Column chromatography was performed using Teledyne ISCO’s RediSep R_f_^®^ pre-loaded silica gel cartridges on a Biotage (Uppsala, Sweden) automated purification system. NMR spectra were collected in DMSO-*d*_6_ on Bruker 400 MHz and 500 MHz spectrometers. All chemical shifts are reported in parts per million (ppm, *δ* units) and are referenced to the residual protons in the deuterated solvent. Coupling constant units are in hertz (Hz). Splitting patterns are indicated as follows: s (singlet), d (doublet), t (triplet), q (quartet), m (multiplet), dd (doublet of doublets), dt (doublet of triplets), td (triplet of doublets), ddd (doublet of doublets of doublets). Water suppressed ^1^H NMR spectra were recorded at 298 K on a Bruker Avance Neo 600 MHz NMR spectrometer equipped with a QCI-F cryoprobe using a solvent suppression pulse sequence with pre-saturation and spoil gradients (1D spectra, noesygppr1d, Bruker (Billerica, MA, USA)) and chemical shifts (*δ* units) referenced to the residual water signal at 4.7 ppm. HRMS samples were analyzed with a Q Exactive HF-X (ThermoFisher, Bremen, Germany) mass spectrometer. Samples were introduced by a heated electrospray source (HESI) at a flow rate of 10 µL/min. HESI source conditions were set as follows: nebulizer temperature 400 °C, sheath gas (nitrogen) 20 arb, auxiliary gas (nitrogen) 0 arb, sweep gas (nitrogen) 0 arb, capillary temperature 320 °C, RF voltage 45 V. The mass range was set to 100–1000 *m*/*z*. All measurements were recorded at a resolution setting of 120,000. Solutions were analyzed at 0.1 mg/mL or less based on responsiveness to the ESI mechanism. Xcalibur (ThermoFisher, Breman, Germany) was used to analyze the data. Molecular formula assignments were determined with the Molecular Formula Calculator (v 1.3.0). All observed species were singly charged, as verified by the unit *m*/*z* separation between mass spectral peaks corresponding to the ^12^C and ^13^C^12^Cc-1 isotopes for each elemental composition. Analytical LCMS data were obtained using a Waters Acquity UPLC system equipped with a photodiode array detector using the following method: solvent A = water + 0.2% FA, solvent B = ACN + 0.1% FA, flow rate = 1 mL/min. The gradient started at 95% A for 0.05 min. Afterwards, it was ramped to 100% B over 2 min and held for an additional minute at this concentration before returning to 95% A. For extended LCMS runs, separations were conducted on an Agilent 1290 Infinity II LC System using an Agilent Infinity Lab PoroShell 120 EC-C18 column (30 °C, 2.7 μm, 2.1 × 50 mm). LC conditions were set at 95% water with 0.1% formic acid (A), ramped linearly over 15.1 min to 100% acetonitrile with 0.1% formic acid (B), and held until 15.3 min. At 15.4 min, the gradient was switched back to 95% A and allowed to re-equilibrate until 18.0 min. The injection volume for all samples was 4 µL. Preparative HPLC was performed using an Agilent 1260 Infinity II LC System equipped with a Phenomenex C18 column (PhenylHexyl, 30 °C, 5 μm, 75 × 30 mm) using the following method: Solvent A: water + 0.05% TFA; Solvent B: acetonitrile; flow rate: 30.00 mL/min. LC conditions were set at 95% A ramped linearly over 26 min to 100% B and held until 28 min at 100% B. At 30 min, the gradient was switched back to 95% A. The final compounds were determined to have ≥95% purity by analytical LCMS.

### 3.2. Optimized Synthesis Pyrazole Vinyl Sulfones (***1***, ***7***) and Dihydropyrazolo[1,5-a]pyrazin-4(5H)-ones (***2***, ***8***)

#### 3.2.1. (*E*)-5-(2-Ethoxyphenyl)-*N*-(3-(methylsulfonyl)allyl)-1*H*-pyrazole-3-carboxamide (**1**)

Method A: To a stirred solution of 5-(2-ethoxyphenyl)-1*H*-pyrazole-3-carboxylic acid (**3**, 100 mg, 0.43 mmol, 1.0 eq.) and TBTU (207 mg, 0.65 mmol, 1.5 eq.) in pyridine (3 mL), (*E*)-3-(methylsulfonyl)prop-2-en-1-amine (**4**, 89 mg, 0.52 mmol, 1.2 eq.) was added, and the reaction was stirred at 25 °C for 2 h. On completion of the reaction based on TLC and LCMS analysis, the reaction was poured into water and extracted with EtOAc. The combined organic layers were dried over anhydrous Na_2_SO_4_, filtered, and concentrated to give the crude compound. Column chromatography (eluting with 0–100% EtOAc in hexanes) followed by preparative HPLC purification afforded the TFA salt of (*E*)-5-(2-ethoxyphenyl)-*N*-(3-(methylsulfonyl)allyl)-1*H*-pyrazole-3-carboxamide (**1**) as a white solid (140 mg, 70%): m.p. 70 °C; ^1^H NMR (DMSO-*d*_6_, 500 MHz): *δ* 8.62 (t, *J* = 5.9 Hz, 1H), 7.74 (dd, *J* = 7.6, 1.8 Hz, 1H), 7.34 (ddd, *J* = 8.5, 7.4, 1.7 Hz, 1H), 7.16–7.10 (m, 2H), 7.03 (td, *J* = 7.5, 1.1 Hz, 1H), 6.82 (dt, *J* = 15.2, 4.4 Hz, 1H), 6.69 (dt, *J* = 15.2, 1.8 Hz, 1H), 4.16 (q, *J* = 6.9 Hz, 2H), 4.11 (ddd, *J* = 6.1, 4.4, 1.8 Hz, 2H), 3.01 (s, 3H), 1.41 (t, *J* = 6.9 Hz, 3H); ^13^C NMR (DMSO-*d*_6_, 126 MHz): *δ* 161.5, 155.0, 143.5, 130.0, 129.7, 127.8, 120.6, 112.8, 105.6, 63.7, 42.2, 38.7, 14.6; HRMS (ESI) *m*/*z*: [M + H]^+^ calculated for C_16_H_20_N_3_O_4_S: 350.1175; found 350.1172; HPLC purity > 99%.

Method B: To a stirred solution of 5-(2-ethoxyphenyl)-1*H*-pyrazole-3-carboxylic acid (**3**, 1.0 g, 4.3 mmol, 1.0 eq.) in DMSO (10 mL) at 0 °C, K_2_CO_3_ (1.8 g, 13 mmol, 3.0 eq.) and chloromethyl methyl ether (0.39 mL, 5.2 mmol, 1.2 eq.) were added, and the reaction was stirred at 25 °C for 1 h. On completion of the reaction based on TLC and LCMS analysis, the reaction was poured into water and extracted with Et_2_O. The combined organic layers were washed with brine, dried over anhydrous Na_2_SO_4_, filtered, and concentrated to give the crude compound. Column chromatography (eluting with 10% MeOH in CH_2_Cl_2_) afforded 5-(2-ethoxyphenyl)-1-(methoxymethyl)-1*H*-pyrazole-3-carboxylic acid (**5**) as a white solid (350 mg, 29%); ^1^H NMR (DMSO-*d*_6_, 500 MHz): *δ* 13.52 (s, 1H), 7.93 (dd, *J* = 7.7, 1.8 Hz, 1H), 7.37 (s, 1H), 7.33 (ddd, *J* = 8.3, 7.3, 1.8 Hz, 1H), 7.11 (dd, *J* = 8.4, 1.1 Hz, 1H), 7.01 (td, *J* = 7.4, 1.1 Hz, 1H), 5.77 (s, 2H), 4.15 (q, *J* = 6.9 Hz, 2H), 3.28 (s, 3H), 1.41 (t, *J* = 7.0 Hz, 3H); ^13^C NMR (DMSO-*d*_6_, 126 MHz): *δ* 160.4, 155.8, 146.7, 134.0, 129.6, 127.7, 120.5, 120.3, 112.9, 112.8, 80.2, 63.6, 56.3, 14.7; *m*/*z* [M + H] ^+^ 277.

To a stirred solution of **5** (200 mg, 724 μmol, 1.0 eq.) and TBTU (349 mg, 1.09 mmol, 1.5 eq.) in pyridine (5.0 mL), (*E*)-3-(methylsulfonyl) prop-2-en-1-amine (**4**, 149 mg, 869 μmol, 1.2 eq.) was added, and the reaction was stirred at 25 °C for 2 h. On completion of the reaction based on TLC and LCMS analysis, the reaction was poured into water and extracted with EtOAc. The combined organic layers were dried over anhydrous Na_2_SO_4_, filtered, and concentrated to give the crude compound. Column chromatography (eluting with 0–100% EtOAc in hexanes) afforded (*E*)-5-(2-ethoxyphenyl)-1-(methoxymethyl)-*N*-(3-(methylsulfonyl)allyl)-1*H*-pyrazole-3-carboxamide (**6**) as a white solid (210 mg, 74%); ^1^H NMR (DMSO-*d*_6_, 500 MHz): *δ* 9.00 (t, *J* = 5.7 Hz, 1H), 7.90 (dd, *J* = 7.7, 1.8 Hz, 1H), 7.46 (s, 1H), 7.33 (ddd, *J* = 8.2, 7.3, 1.8 Hz, 1H), 7.12 (dd, *J* = 8.5, 1.1 Hz, 1H), 7.00 (td, *J* = 7.5, 1.1 Hz, 1H), 6.82 (dt, *J* = 15.3, 4.2 Hz, 1H), 6.74 (dt, *J* = 15.2, 1.6 Hz, 1H), 5.79 (s, 2H), 4.20–4.11 (m, 4H), 3.27 (s, 3H), 3.01 (s, 3H), 1.44 (t, *J* = 6.9 Hz, 3H); ^13^C NMR (DMSO-*d*_6_, 126 MHz): *δ* 159.2, 155.7, 146.6, 142.9, 136.0, 130.3, 129.5, 127.9, 120.8, 120.5, 112.9, 109.5, 79.9, 63.7, 56.3, 42.2, 38.9, 14.6; *m*/*z* [M + H]^+^ 394.

To a stirred solution of **6** (100 mg, 254 μmol, 1.0 eq.) in CH_2_Cl_2_ was added 4M HCl in dioxane (1.3 mL, 5.1 mmol, 20.0 eq.), and the reaction was stirred at 25 °C for 3 h. On completion of the reaction based on TLC and LCMS analysis, the reaction was concentrated. The resulting solid was washed with Et_2_O and dried under high vacuum to obtain the HCl salt of (*E*)-5-(2-ethoxyphenyl)-*N*-(3-(methylsulfonyl)allyl)-1*H*-pyrazole-3-carboxamide (**1**) as a white solid (90 mg, 92%); m.p. 198 °C; HPLC purity > 99%.

#### 3.2.2. 2-(2-Ethoxyphenyl)-7-((methylsulfonyl)methyl)-6,7-dihydropyrazolo[1,5-*a*]pyrazin-4(5H)-one (**2**)

To a stirred solution of (*E*)-5-(2-ethoxyphenyl)-*N*-(3-(methylsulfonyl)allyl)-1*H*-pyrazole-3-carboxamide (**1**, 100 mg, 286 μmol, 1.0 eq.) in 1,4-dioxane (1.5 mL) and water (1.5 mL), sodium carbonate (91.0 mg, 859 μmol, 3.0 eq.) was added, and the reaction was stirred at 25 °C for 12 h. On completion of the reaction based on LCMS analysis, the reaction was poured into water and extracted with EtOAc. The combined organic layers were dried over anhydrous Na_2_SO_4_, filtered, and concentrated to give the crude product. Column chromatography (eluting with 0–100% EtOAc in hexanes) afforded 2-(2-ethoxyphenyl)-7-((methylsulfonyl)methyl)-6,7-dihydropyrazolo[1,5-*a*]pyrazin-4(5*H*)-one (**2**) as a white solid (70 mg, 69%): m.p. 228 °C; ^1^H NMR (DMSO-*d*_6_, 500 MHz): *δ* 8.31 (t, *J* = 2.9 Hz, 1H), 7.95 (dd, *J* = 7.7, 1.8 Hz, 1H), 7.32 (ddd, *J* = 8.9, 7.4, 1.8 Hz, 1H), 7.23 (s, 1H), 7.11 (dd, *J* = 8.4, 1.0 Hz, 1H), 7.00 (td, *J* = 7.5, 1.0 Hz, 1H), 5.07 (dq, *J* = 9.0, 4.5 Hz, 1H), 4.15 (q, *J* = 7.0 Hz, 2H), 3.98 (dd, *J* = 14.4, 3.9 Hz, 1H), 3.93 (ddd, *J* = 13.3, 4.5, 2.4 Hz, 1H), 3.76–3.68 (m, 2H), 3.16 (s, 3H), 1.41 (t, *J* = 6.9 Hz, 3H); ^13^C NMR (DMSO-*d*_6_, 126 MHz): *δ* 158.1, 158.1, 155.7, 147.3, 134.8, 129.4, 127.8, 120.5, 120.4, 112.7, 107.9, 63.6, 53.8, 50.9, 43.0, 41.5, 14.7; HRMS (ESI) *m*/*z*: [M + H]^+^ calculated for C_16_H_20_N_3_O_4_S: 350.1175; found 350.1177; HPLC purity > 99%.

#### 3.2.3. (*E*)-*N*-(3-(Methylsulfonyl)allyl)-5-(phenylsulfonamido)-1*H*-pyrazole-3-carboxamide (**7**)

To a stirred solution of 5-(phenylsulfonamido)-1*H*-pyrazole-3-carboxylic acid (184 mg, 688 μmol, 1.0 eq.) and TBTU (332 mg, 1.03 mmol, 1.5 eq.) in pyridine (5.0 mL), (*E*)-3-(methylsulfonyl) prop-2-en-1-amine (**4**, 142 mg, 0.83 mmol, 1.2 eq.) was added, and the reaction was stirred at 25 °C for 2 h. On completion of the reaction based on TLC and LCMS analysis, the mixture was poured into water and extracted with EtOAc. The combined organic layers were dried over anhydrous Na_2_SO_4_, filtered, and concentrated to give the crude compound. Column chromatography (eluting with 0–100% EtOAc in hexanes), followed by preparative HPLC purification afforded the TFA salt of (*E*)-*N*-(3-(methylsulfonyl)allyl)-5-(phenylsulfonamido)-1*H*-pyrazole-3-carboxamide (**7**) as a white solid (125 mg, 36%): m.p. 181 °C; ^1^H NMR (DMSO-*d*_6_, 500 MHz): *δ* 13.17 (s, 1H), 10.64 (s, 1H), 8.86 (s, 1H), 7.80–7.77 (m, 2H), 7.63 (t, *J* = 7.3 Hz, 1H), 7.57 (t, *J* = 7.5 Hz, 2H), 6.82–6.69 (m, 3H), 4.07 (t, *J* = 4.7 Hz, 2H), 3.00 (s, 3H); ^13^C NMR (DMSO-*d_6_*, 126 MHz): δ 158.4, 145.9, 142.7, 140.1, 136.9, 132.8, 130.3, 129.1, 126.6, 97.3, 42.1, 38.7; HRMS (ESI) *m*/*z*: [M + H]^+^ calculated for C_14_H_17_N_4_O_5_S_2_ 385.0640; found 385.0577; HPLC purity > 98%.

#### 3.2.4. *N*-(7-((Methylsulfonyl)methyl)-4-oxo-4,5,6,7-tetrahydropyrazolo[1,5-*a*]pyrazin-2-yl)benzenesulfonamide (**8**)

To a stirred solution of (*E*)-*N*-(3-(methylsulfonyl)allyl)-5-(phenylsulfonamido)-1*H*-pyrazole-3-carboxamide 2,2,2-trifluoroacetate (**7**, 50.0 mg, 100 μmol, 1.0 eq.) in 1,4-dioxane (1.5 mL) and water (1.5 mL) NaHCO_3_ (25 mg, 300 μmol, 3.0 eq.) was added, and the reaction was stirred at 25 °C for 12 h. On completion of the reaction based on LCMS analysis, the mixture was poured into water and extracted with EtOAc. The combined organic extracts were dried over anhydrous Na_2_SO_4_, filtered, and concentrated to give the crude compound. Column chromatography (eluting with 0–100% EtOAc in hexanes), followed by preparative HPLC purification afforded the TFA salt of *N*-(7-((methylsulfonyl)methyl)-4-oxo-4,5,6,7-tetrahydropyrazolo[1,5-*a*]pyrazin-2-yl)benzenesulfonamide (**8**) as a white solid (20.0 mg, 39%): m.p 222 °C; ^1^H NMR (DMSO-*d*_6_, 500 MHz): *δ* 10.97 (s, 1H), 8.29 (t, *J* = 2.8 Hz, 1H), 7.83–7.81 (m, 2H), 7.67–7.63 (m, 1H), 7.60–7.56 (m, 2H), 6.36 (d, *J* = 1.3 Hz, 1H), 4.82 (dq, *J* = 9.2, 4.7 Hz, 1H), 3.81 (ddd, *J* = 13.4, 4.5, 2.2 Hz, 1H), 3.65 (dd, *J* = 14.5, 4.6 Hz, 1H), 3.62–3.56 (m, 2H), 2.99 (s, 3H); ^13^C NMR (DMSO-*d*_6_, 176 MHz): *δ* 157.3, 145.9, 139.9, 135.0, 133.0, 129.3, 126.7, 97.9, 53.9, 50.5, 42.9, 41.4; ^13^C NMR (DMSO-*d*_6_, 214 MHz): *δ* 157.3, 145.9, 139.9, 135.0, 133.1, 129.3, 126.7, 97.9, 53.9, 50.5, 42.9, 41.5. HRMS (ESI) *m*/*z*: [M + H]^+^ calculated for C_14_H_17_N_4_O_5_S_2_ 385.0640; found 385.0584; HPLC purity > 98%.

### 3.3. ^1^H NMR Stability Assay

A 40 mM stock solution of **1** in DMSO-*d_6_* was prepared. A 95 mM stock solution of maleic acid was prepared in D_2_O. The stock solutions of **1** (27.5 μL, 1.1 μmol) and maleic acid (5.8 μL, 0.55 μmol), were added to D_2_O (49.2 μL) and pH 7.4 phosphate buffer (467.5 μL, 200 mM). The mixture was analyzed periodically by ^1^H NMR (600 MHz, H_2_O/D_2_O 9:1, noesygppr1d, 256 scans) and held at room temperature between NMR acquisitions.

### 3.4. Pharmacokinetic Methods

Male CD1 mice were dosed intravenously with 10 mg/Kg solutions of **1** in DMSO/PEG-400/Water (*v*/*v*/*v*, 5/40/55) or **2** in NMP/Solutol/PEG-400/normal saline (10:5:40:45; *v*/*v*/*v*/*v*). Blood was collected at intervals of 0.25, 0.5, 1, and 3 h (for **1**) and 0.5, 1, 3, and 5 h (for **2**) post-dose from the dorsal metatarsal vein and transferred into plastic microcentrifuge tubes containing anticoagulant EDTA-K2. Blood samples were centrifuged at 4000× *g* for 5 min at 4 °C to obtain plasma. The plasma samples from each time point were pooled and then analyzed by LCMS/MS. The PK parameters were estimated by a non-compartmental model using WinNonlin 8.3.

### 3.5. GSH Capture Assay

A 10 mM solution of GSH (Sigma Aldrich Cat# G4251 (St. Louis, MO, USA)) was prepared in a pH 7.4 phosphate buffer. A 10 mM DMSO solution of the test compound was diluted in phosphate buffer to give a solution at 100 µM with 1% DMSO. At time zero (t = 0), 50 µL of the 100 µM test compound solution was added to an Eppendorf tube containing 50 µL phosphate buffer and 50 µL of 10 mM GSH solution. The final concentrations of the compound and GSH were maintained at 50 µM and 5 mM, respectively. The Eppendorf tube was vortexed, and then the sample was transferred to a high-recovery autosampler vial for LCMS analysis. Analysis was performed at 8 and 24 h time points, and the percentage of GSH adduct formation was calculated using Agilent LCMS software (OpenLab CDS Version 2.7).

## Figures and Tables

**Table 1:** Optimization of Amide Coupling between **3** and **4** *^a^*.

Entry	Coupling Agent	Base	pK_a_	Solvent	Temp (°C)	Time (h)	Ratio of 1:2 *^b^*
1	HBTU	DIPEA	10.9	DMF	25	16	60:40
2	HATU	DIPEA	10.9	DMF	25	2	60:40
3	PyBOP	DIPEA	10.9	DMF	25	16	60:40
4	T3P	TEA	10.7	DMF	25	2	60:40
5	DIC	DMAP	9.7	DMF	25	16	60:40
6	EDC	HOBt	4.6	MeCN	25	2	70:30
7	TBTU	Pyridine	5.2	Pyridine	25	2	100:0

*^a^* Reaction conditions: **3** (1.0 eq.), **4** (1.2 eq.), solvent (0.2 M). *^b^* Quantified by analytical UPLC analysis.

**Table 2:** Optimization of Intramolecular Cyclization of **1** to **2** *^a^*.

Entry	Base	Eq.	Solvent	Ratio of 1:2 *^b^*
1	K_2_CO_3_	3.5	EtOH	0:100
2	K_2_CO_3_	3.5	H_2_O	100:0
3	Na_2_CO_3_	3.0	Dioxane/H_2_O	0:100
4	NaHCO_3_	3.0	MeOH	12:88
5	NaHCO_3_	3.0	H_2_O	47:53
6	TEA	3.0	MeOH	51:49
7	DIPEA	3.0	DMF	4:96
8	DBU	0.5	ACN	8:92

*^a^* Reaction conditions: 25 °C, 2 h. *^b^* Quantified by analytical UPLC analysis.

**Table 3:** Reversibility of the aza-Michael cyclization by GSH capture of the acyclic vinyl sulfones.

Time (h)	GSH-Adduct Formation (%) *^a^*			
	Acyclic		Cyclic	
	1	7	2	8
8	100	93	0	3
24	100	100	0	6

*^a^* Reaction conditions: GSH (100×), phosphate buffer, 30 °C.

## Data Availability

The original contributions presented in the study are included in the article/Supplementary Material, further inquiries can be directed to the corresponding author.

## Supplementary Materials

The following supporting information can be downloaded at: https://www.mdpi.com/article/10.3390/ph17070836/s1, Figure S1: GSH Capture Spectra. Analytical data for **1**, **2**, **7** and **8**. Figures S2–S28. NMR Spectra. Figures S29–S32. LCMS Spectra. Figures S33–S36. HRMS Spectra. Table S1. nsP2 Protease Inhibition Data.
