# Supplementary material for: Identification of Dihydropyrazolo[1,5-a]pyrazin-4(5H)-ones as Cyclic Products of β-Amidomethyl Vinyl Sulfone Alphavirus Cysteine Protease Inhibitors
Source: Pharmaceuticals (Basel). 2024 Jun 26;17(7):836. doi: 10.3390/ph17070836 (PMC11279629; doi:10.3390/ph17070836)

Supporting Information

Identification of Dihydropyrazolo[1,5-*a*]pyrazin-4(5*H*)-ones as Cyclic Products of β-Amidomethyl Vinyl Sulfone Alphavirus Cysteine Protease Inhibitors

Anirban Ghoshal[1], Álvaro F. Magalhães[2], Kesatebrhan Haile Asressu[1], Mohammad Anwar Hossain[1], Matthew H. Todd[2] and Timothy M. Willson[1]*

[1] Structural Genomics Consortium, UNC Eshelman School of Pharmacy, University of North Carolina at Chapel Hill, Chapel Hill, NC 27599, USA.
[2] Structural Genomics Consortium, Department of Pharmaceutical and Biological Chemistry, University College London School of Pharmacy, London, WC1N 1AX, UK.

* Correspondence: tim.willson@unc.edu

Table of Contents

		Pages
Table S1	nsP2 Protease Inhibition Data	S2
Figure S1	GSH Capture Spectra	S3
Figures S2–S28	NMR Spectra	S4–S17
Figures S29–S32	LCMS Spectra	S18–S19
Figures S33–S36	HRMS Spectra	S20–S21

Table S1: nsP2 Protease Inhibition Data

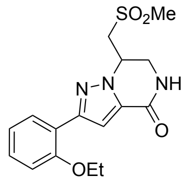

2

Compound	nsP2 Protease (pIC_{50}) [a]
**2**	i.a.

[a]Data from a 10-point dose response curve in triplicate. i.a. = inactive at 200 μM. Assay performed as described in [1].

1. Merten, E.M.; Sears, J.D.; Leisner, T.M.; Hardy, P.B.; Ghoshal, A.; Hossain, M.A.; Asressu, K.H.; Brown, P.J.; Stashko, M.A.; Herring, L.E.; et al. Discovery of a cell-active chikungunya virus nsP2 protease inhibitor using a covalent fragment-based screening approach. *bioRxiv* **2024**, doi:10.1101/2024.03.22.586341.

Figure S1: GSH capture

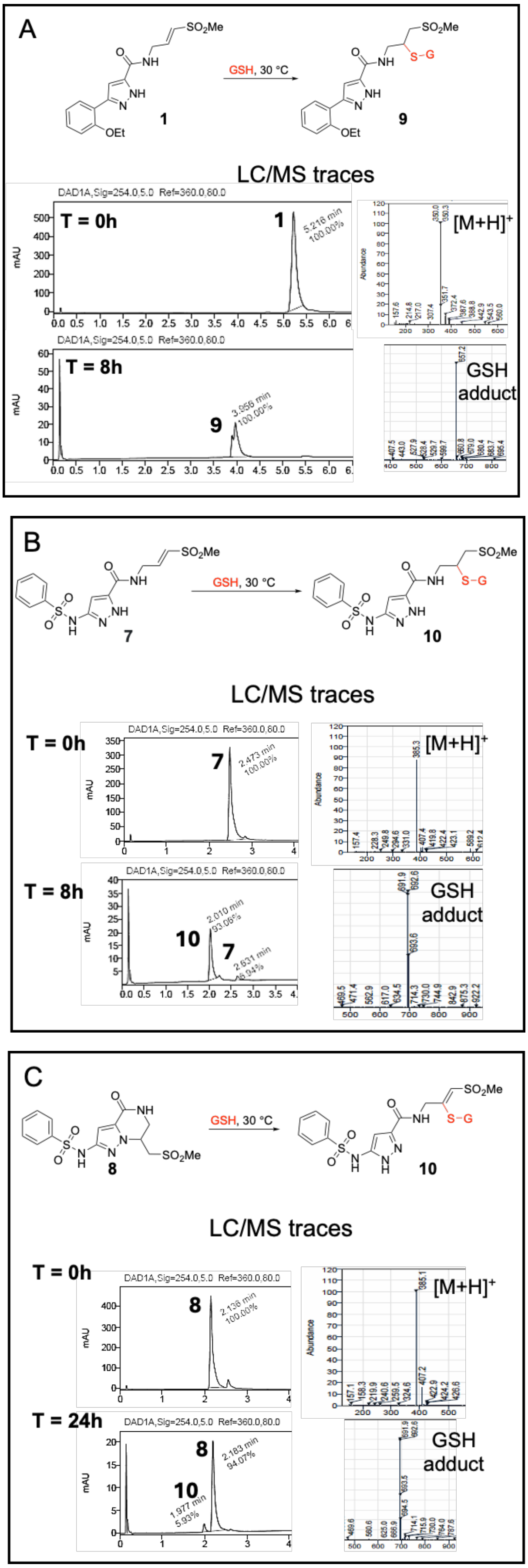

Figure S2: ^{1}H NMR (500 MHz, DMSO-d_6) for **1•TFA**

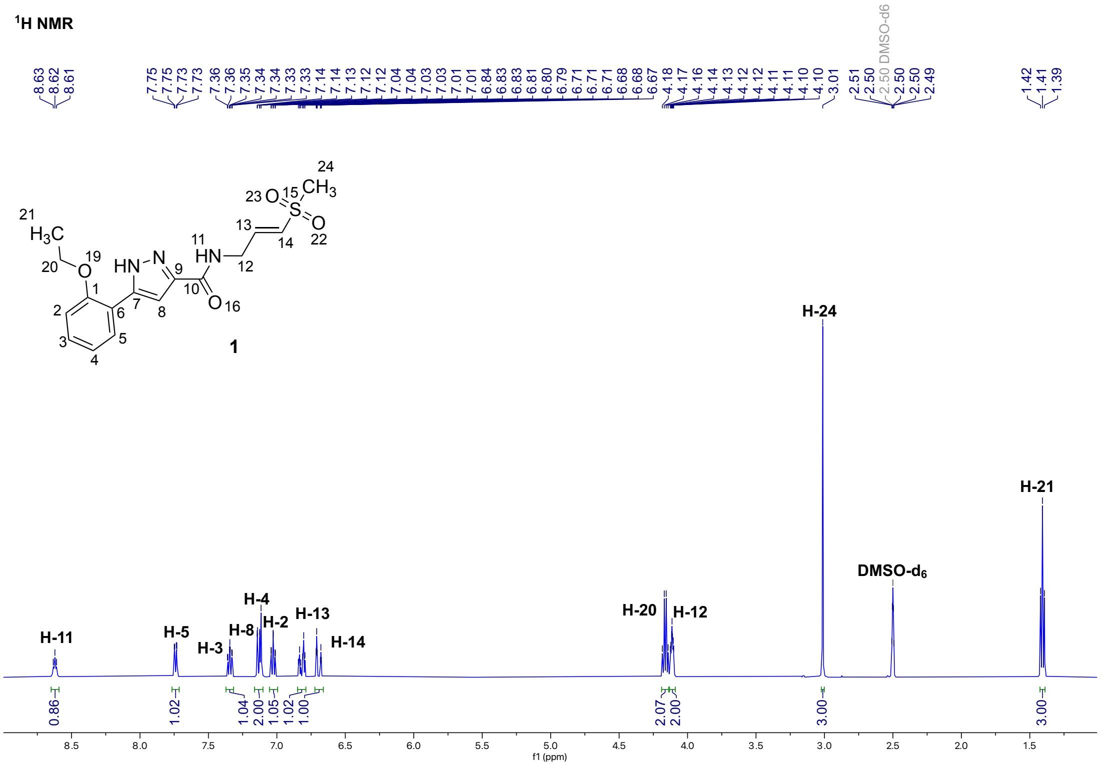

Figure S3: ^{13}C NMR (126 MHz, DMSO-d_6) for **1•TFA**

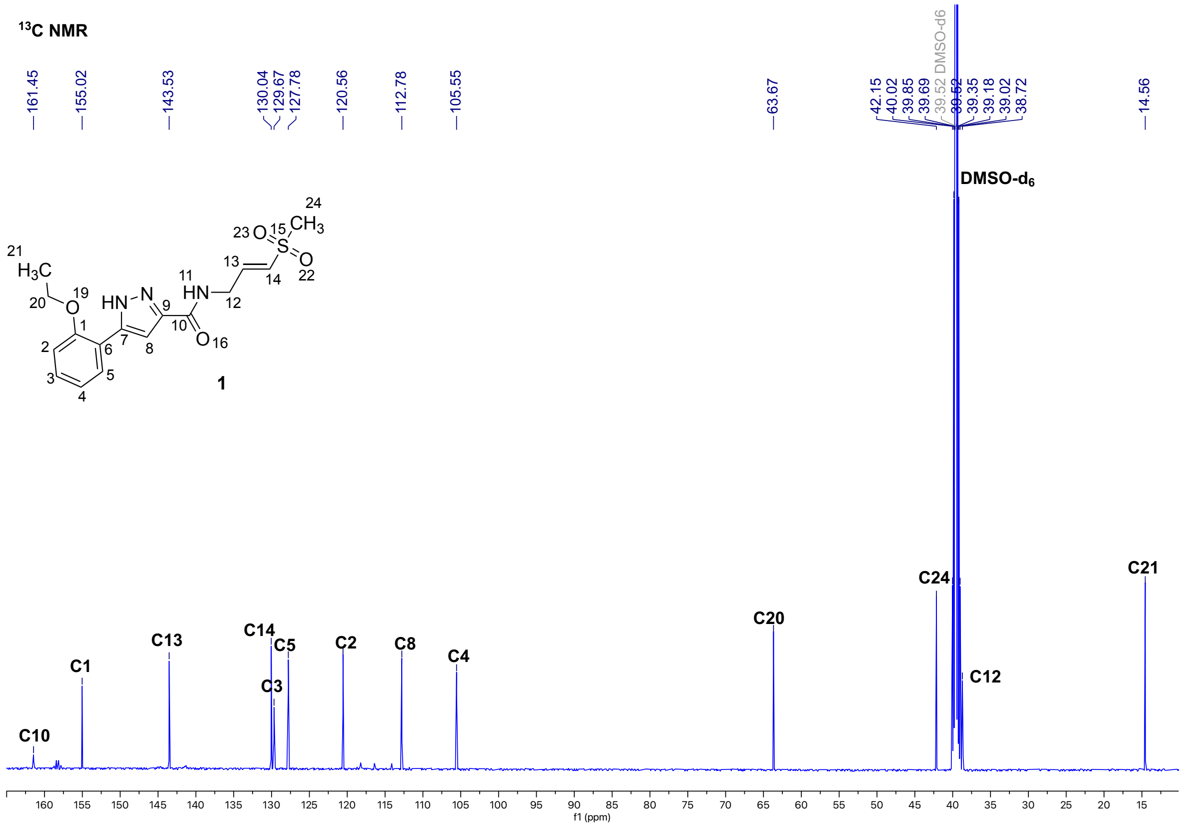

Figure S4: ^{13}C NMR DEPT-135 (126 MHz, DMSO-d_6) for **1•TFA**

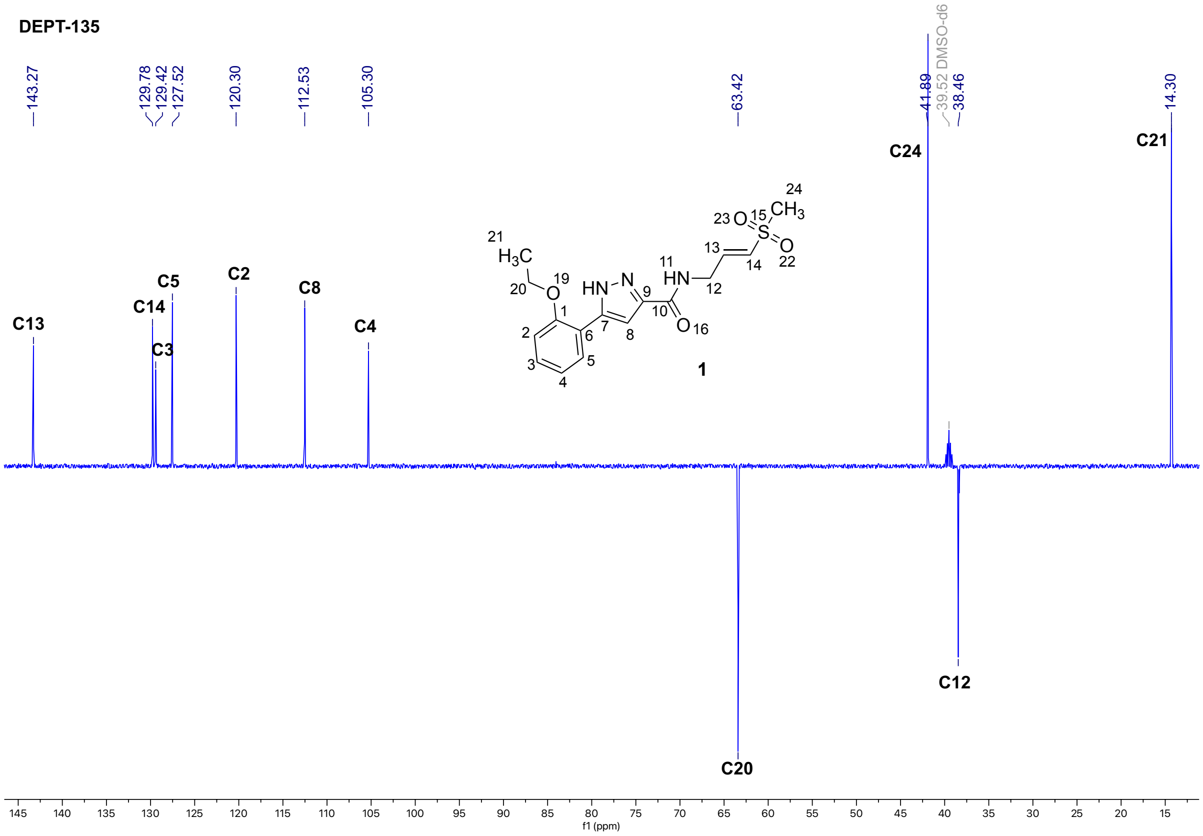

Figure S5: ^{19}F NMR (500 MHz, DMSO-d_6) for **1•TFA**

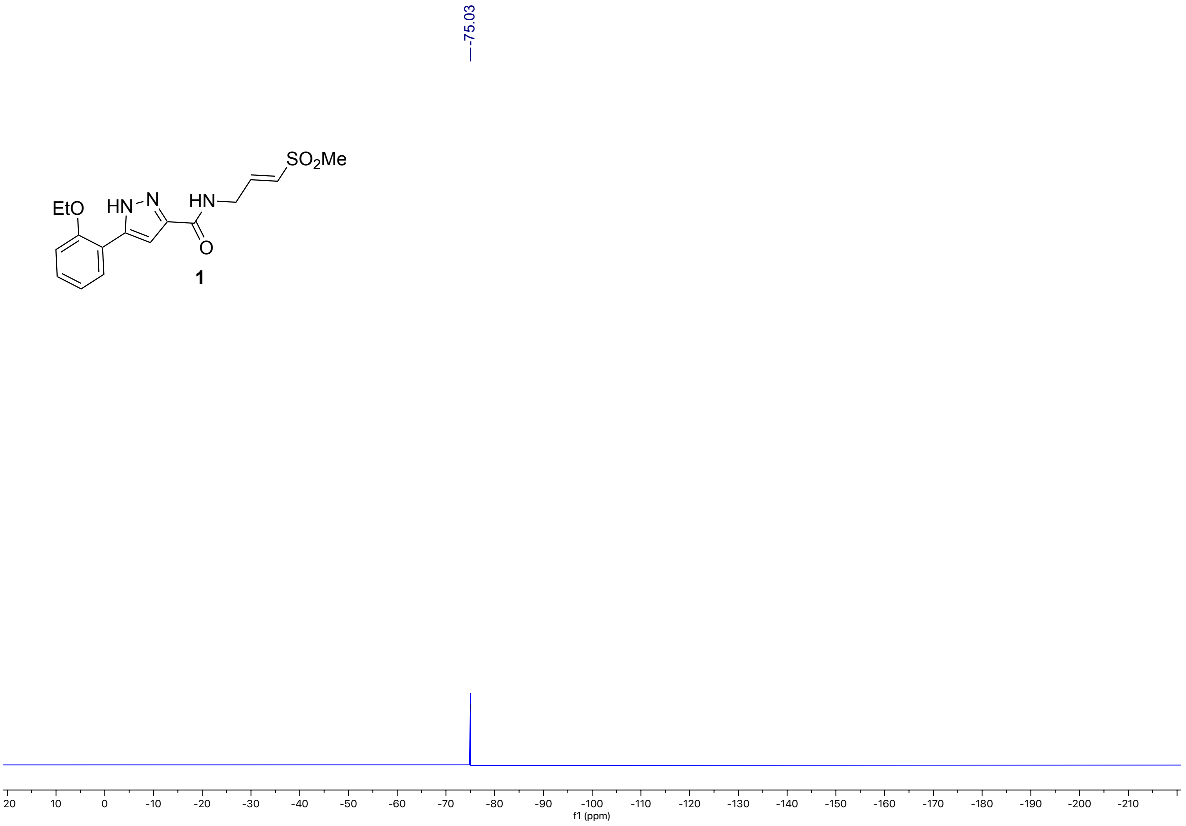

Figure S6: ^{1}H-^{1}H COSY NMR (DMSO-d_6) for **1•TFA**

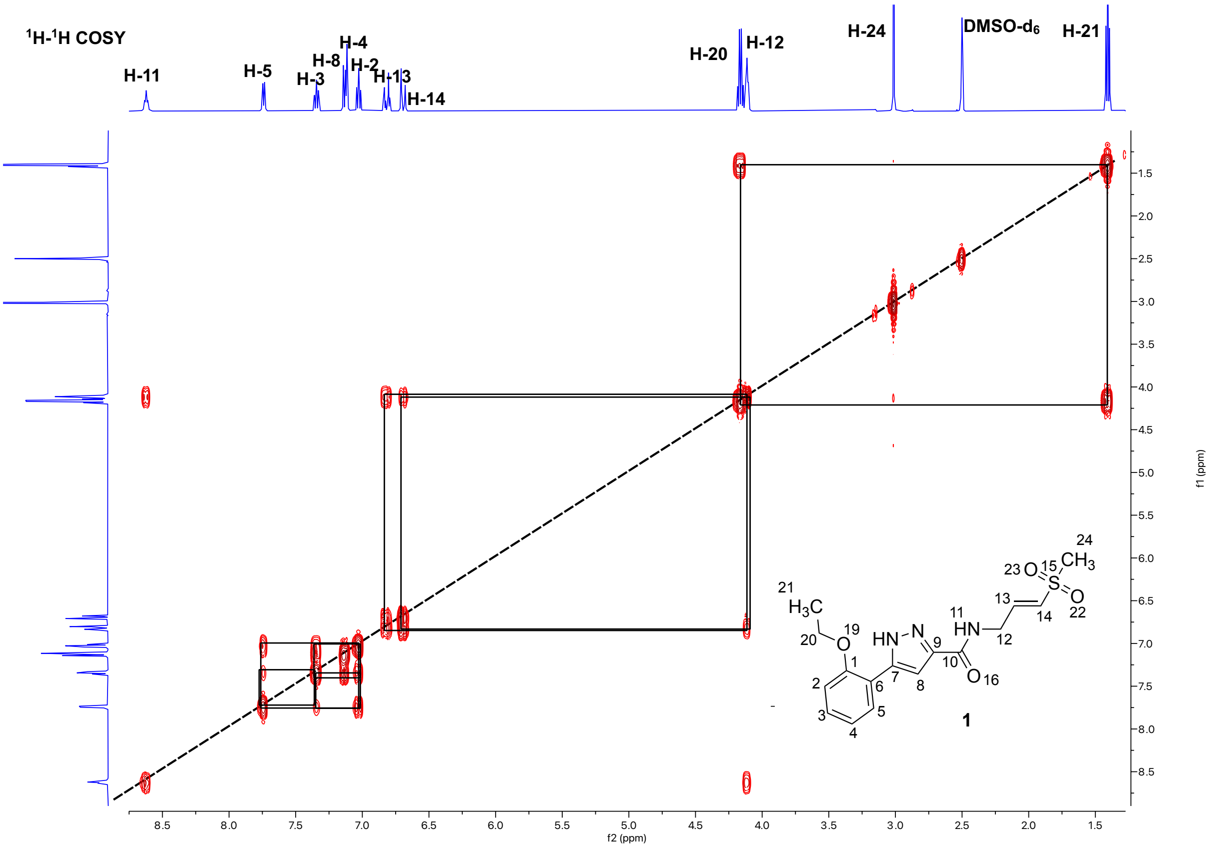

Figure S7: ^{1}H-^{13}C HSQC NMR (DMSO-d_6) for **1•TFA**

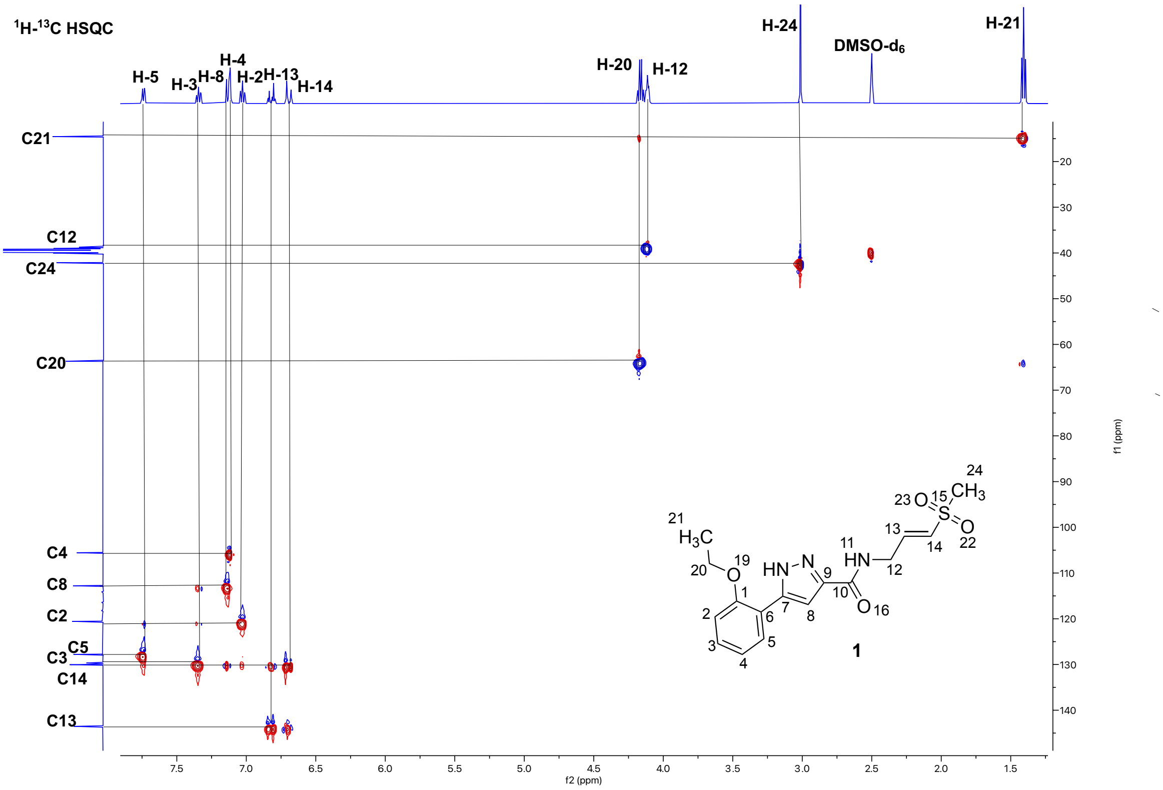

Figure S8: ^{1}H-^{13}C HMBC NMR (DMSO-*d*$_6$) for **1•TFA**

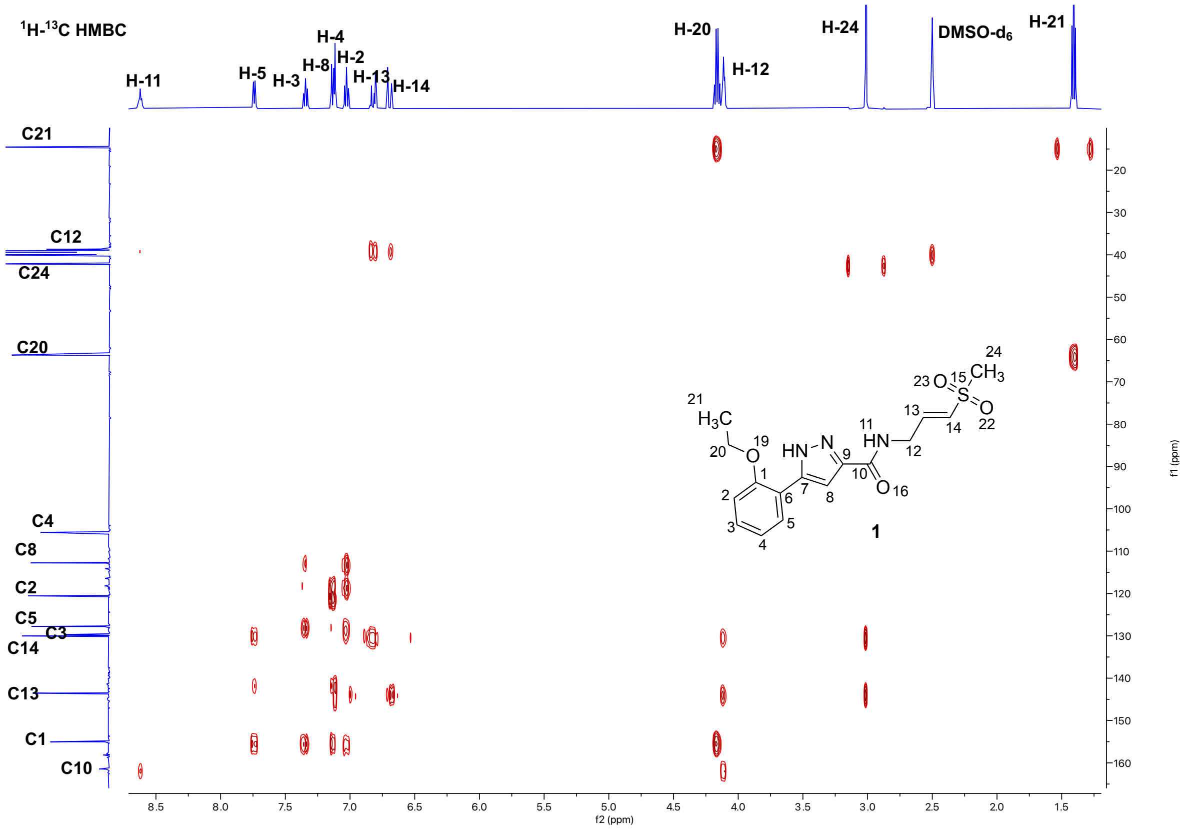

Figure S9: ^{1}H NMR (500 MHz, DMSO-*d*$_6$) for **2**

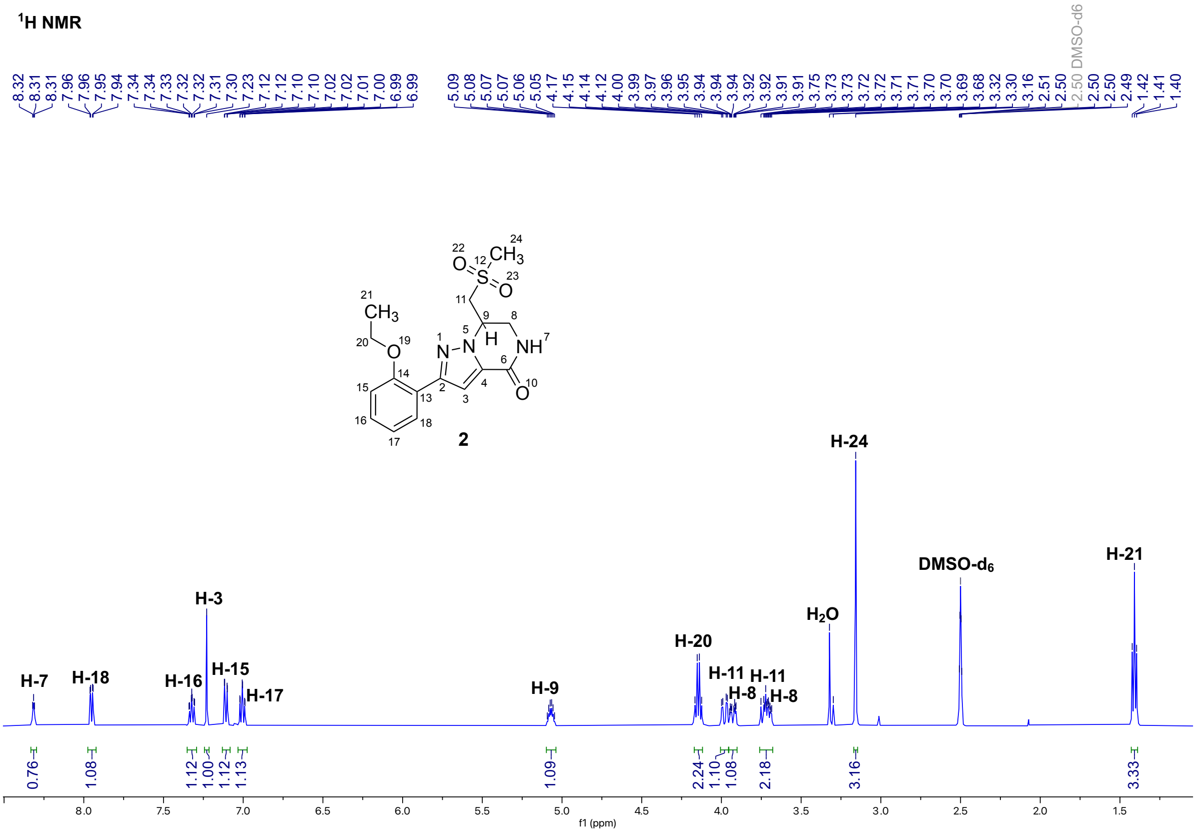

Figure S10: ^{13}C NMR (126 MHz, DMSO-d_6) for **2**

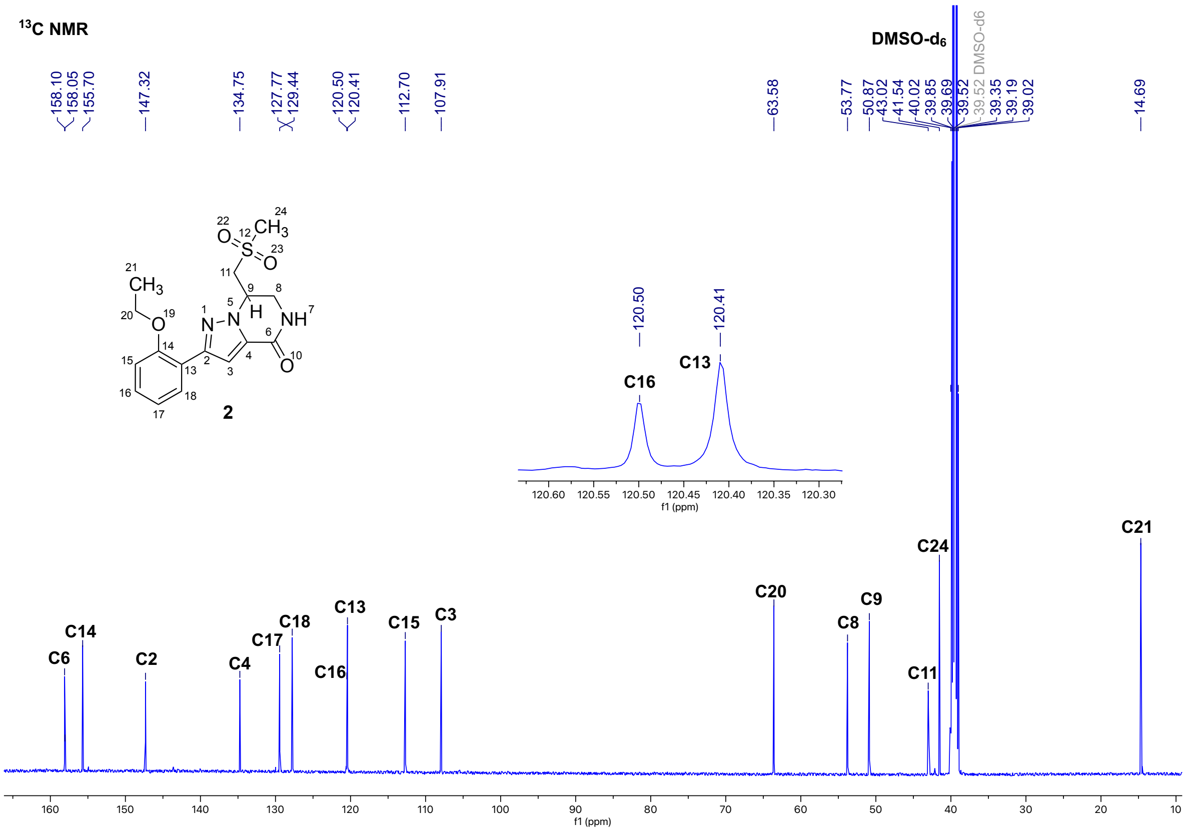

Figure S11: ^{13}C NMR DEPT-135 (126 MHz, DMSO-d_6) for **2**

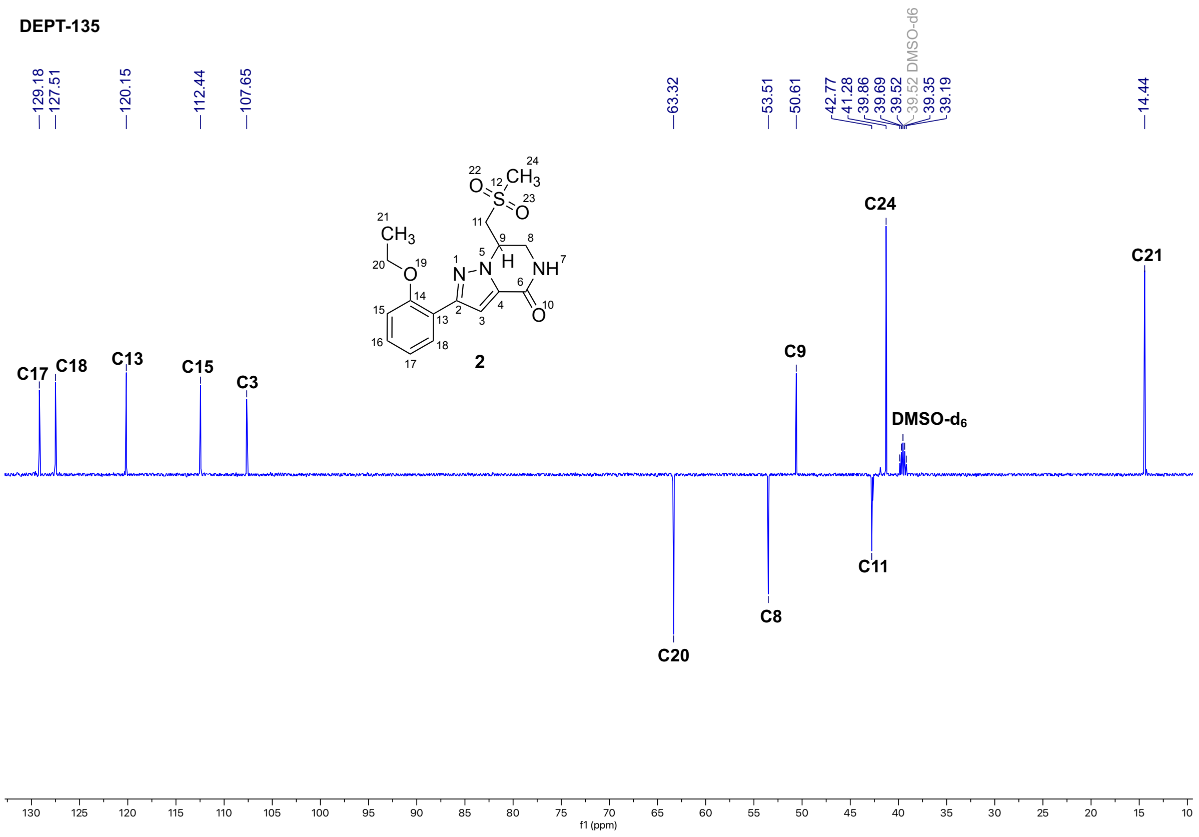

Figure S12: ^{1}H-^{1}H COSY NMR (DMSO-d_6) for **2**

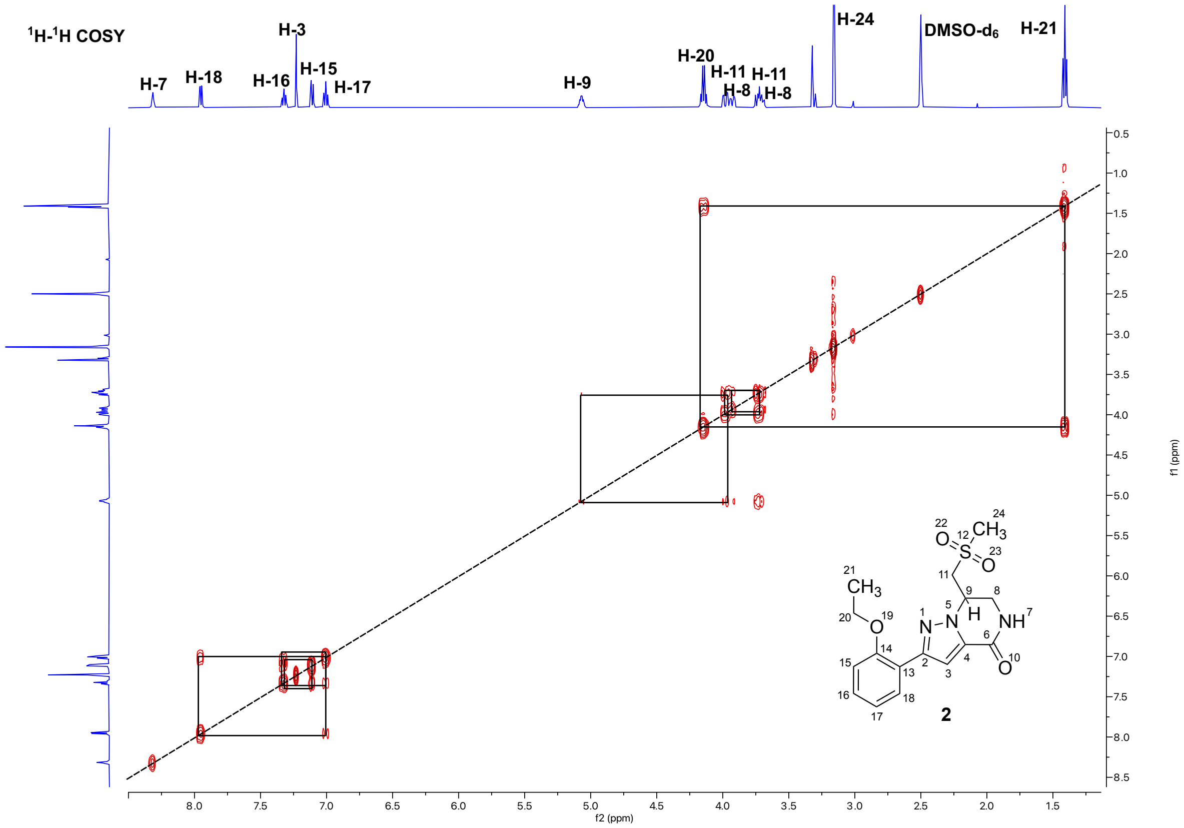

Figure S13: ^{1}H-^{13}C HSQC NMR (DMSO-d_6) for **2**

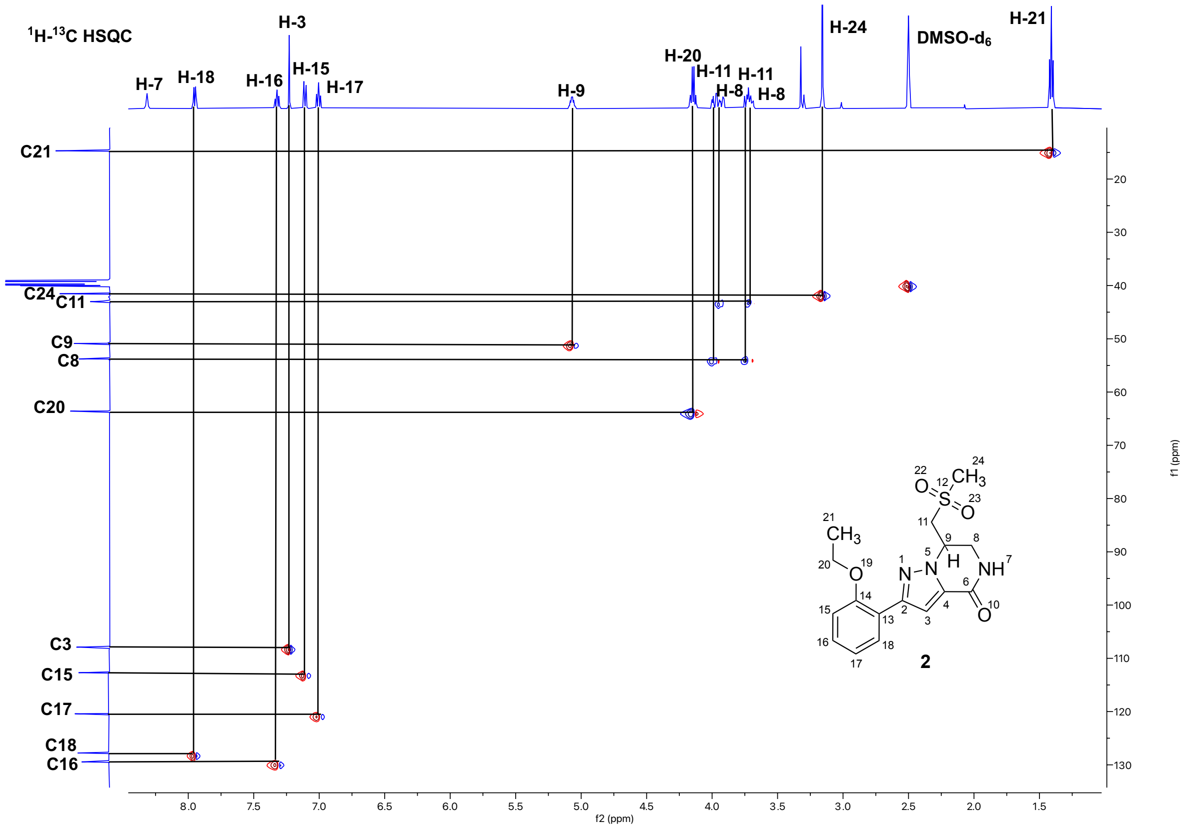

Figure S14: ^{1}H-^{13}C HMBC NMR (DMSO-d_6) for **2**

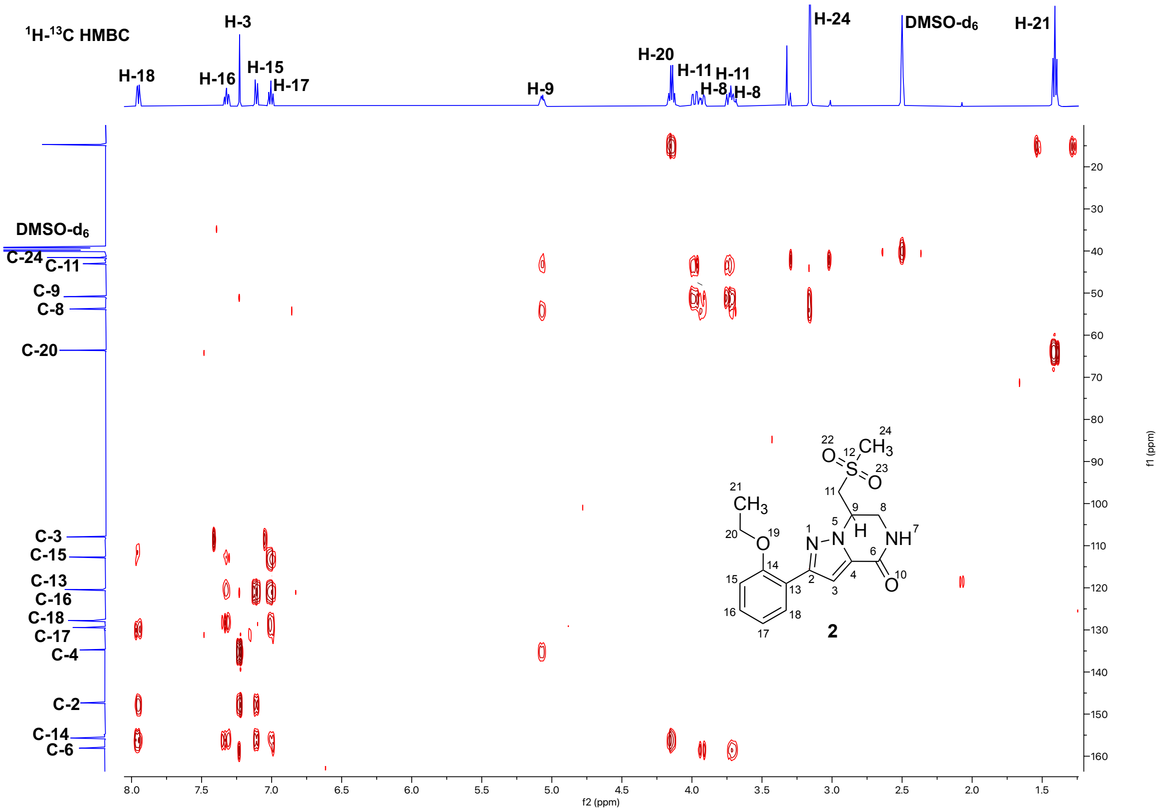

Figure S15: ^{1}H-^{13}C HMBC NMR (DMSO-d_6) of expanded aromatic region for **2**

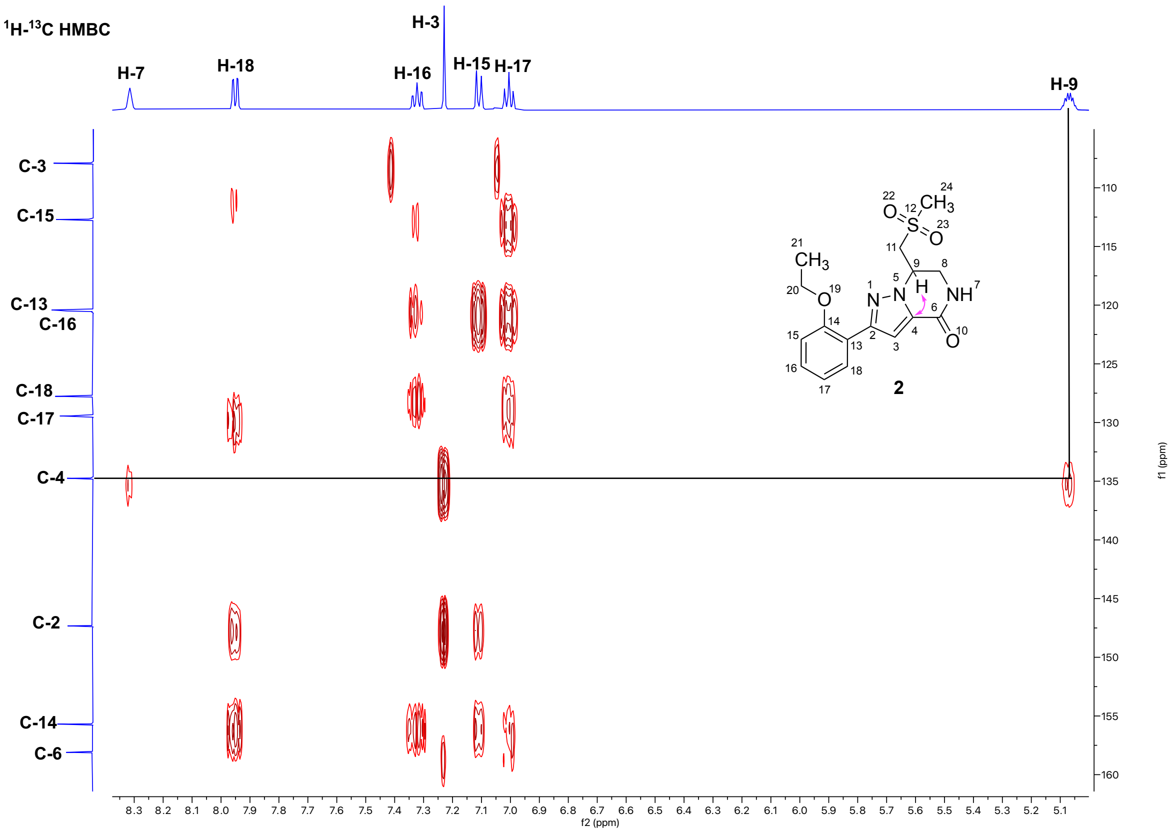

Figure S16: ^{1}H NMR (500 MHz, DMSO-d_6) for **7•TFA**

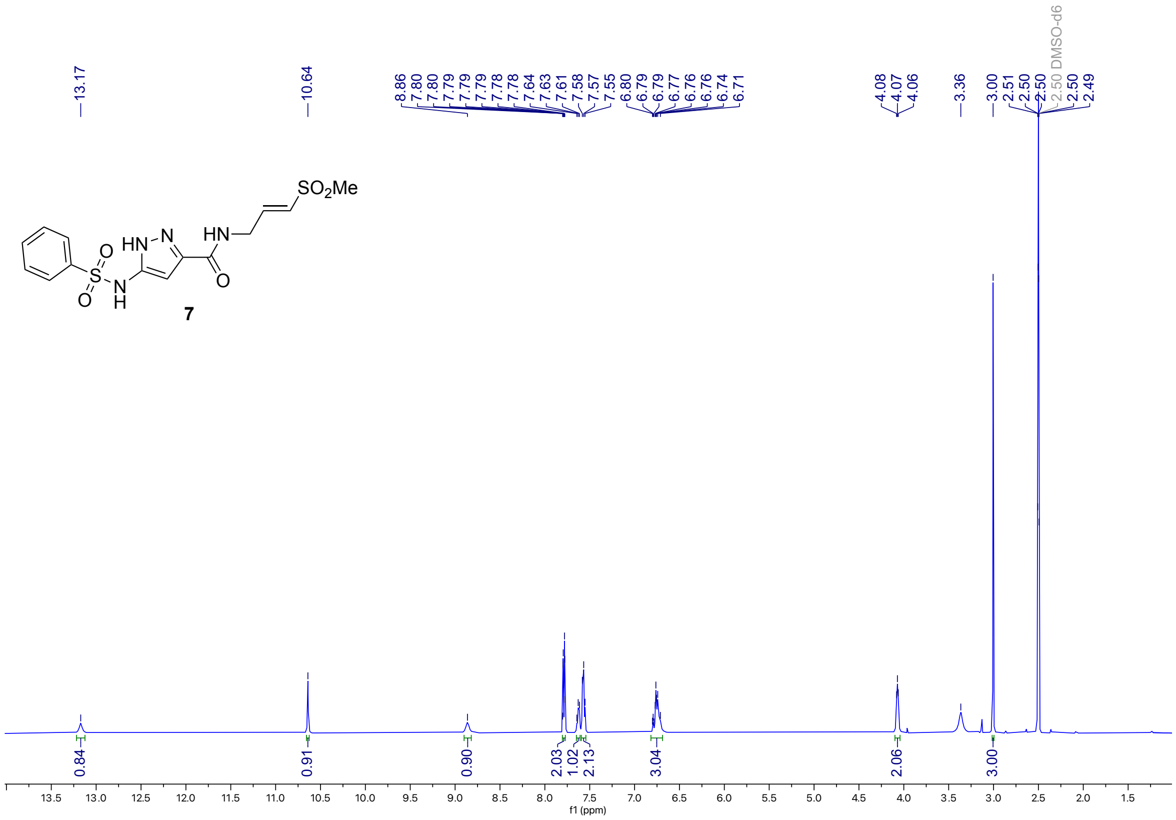

Figure S17: ^{1}H NMR (500 MHz, DMSO-d_6) for **5**

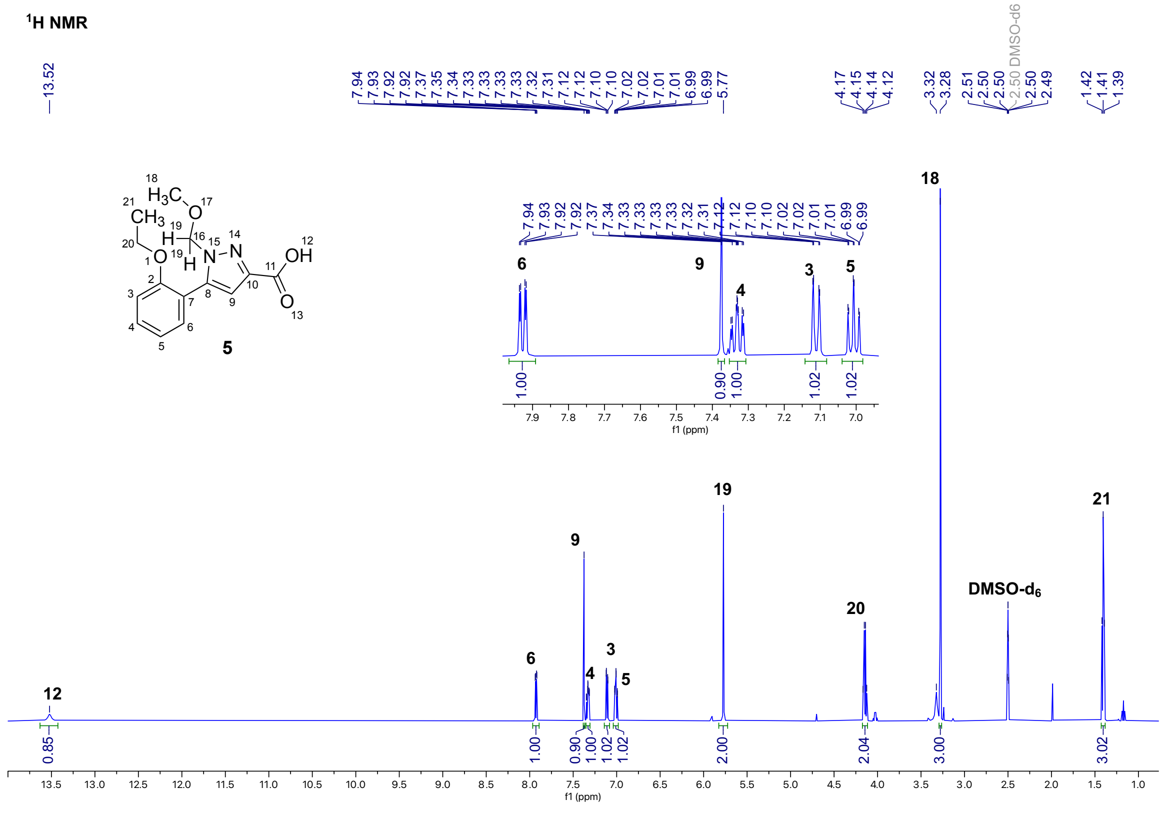

Figure S18: ^{13}C NMR (126 MHz, DMSO-d_6) for **5**

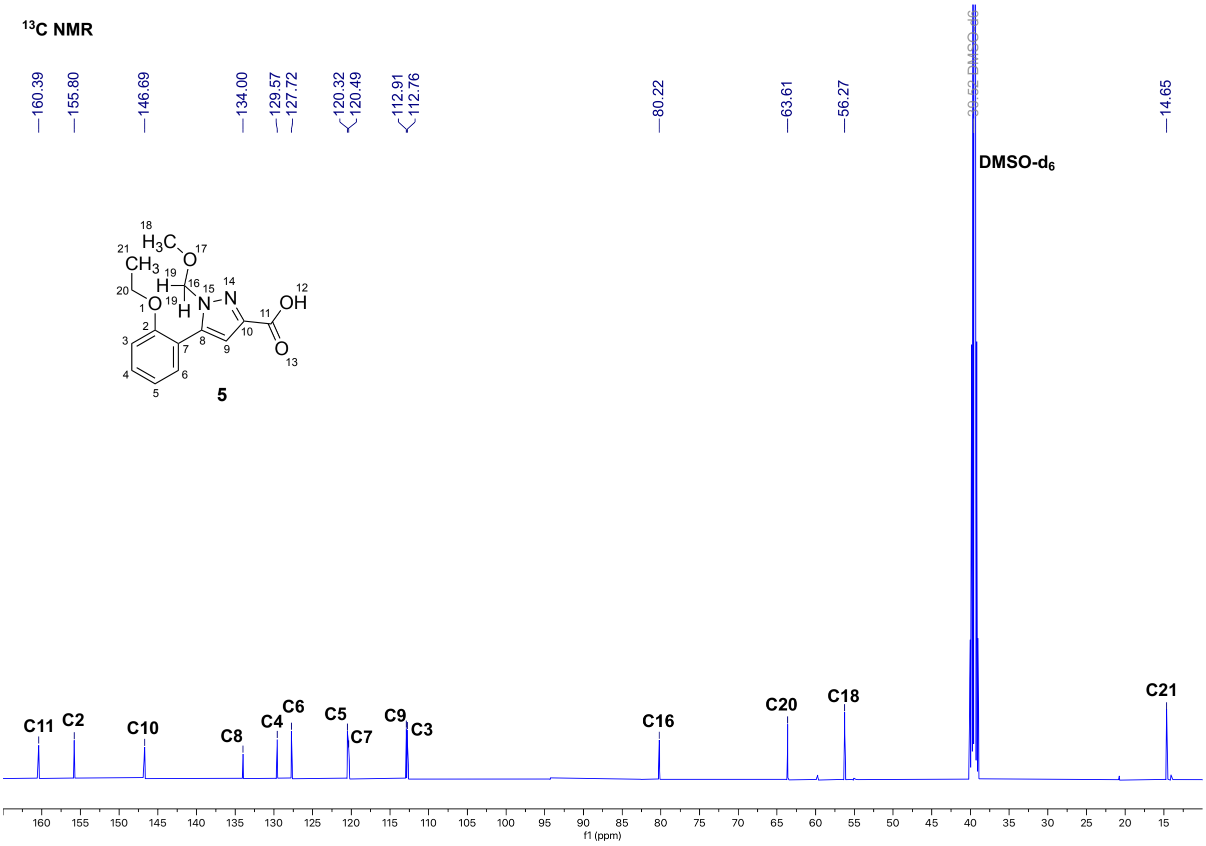

Figure S19: ^{13}C NMR DEPT-135 (126 MHz, DMSO-d_6) for **5**

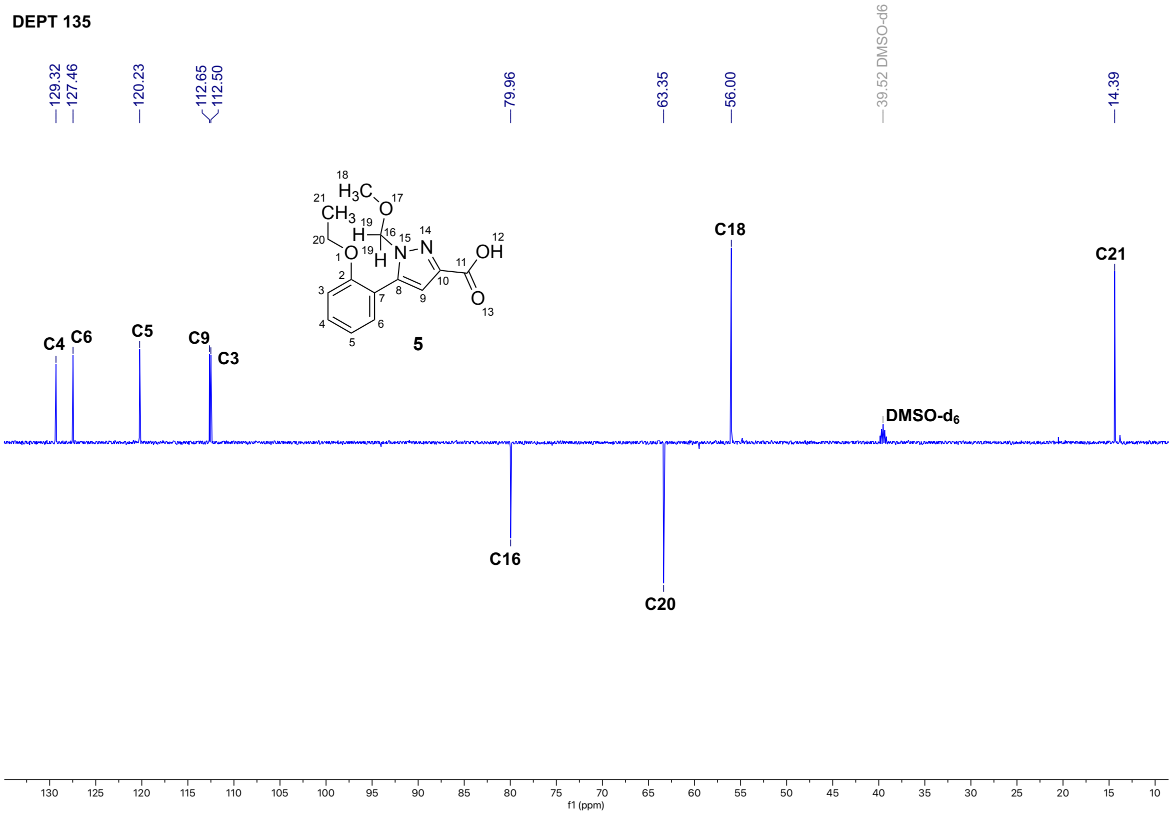

Figure S20: ^{1}H-^{1}H COSY NMR (DMSO-d_6) for **5**

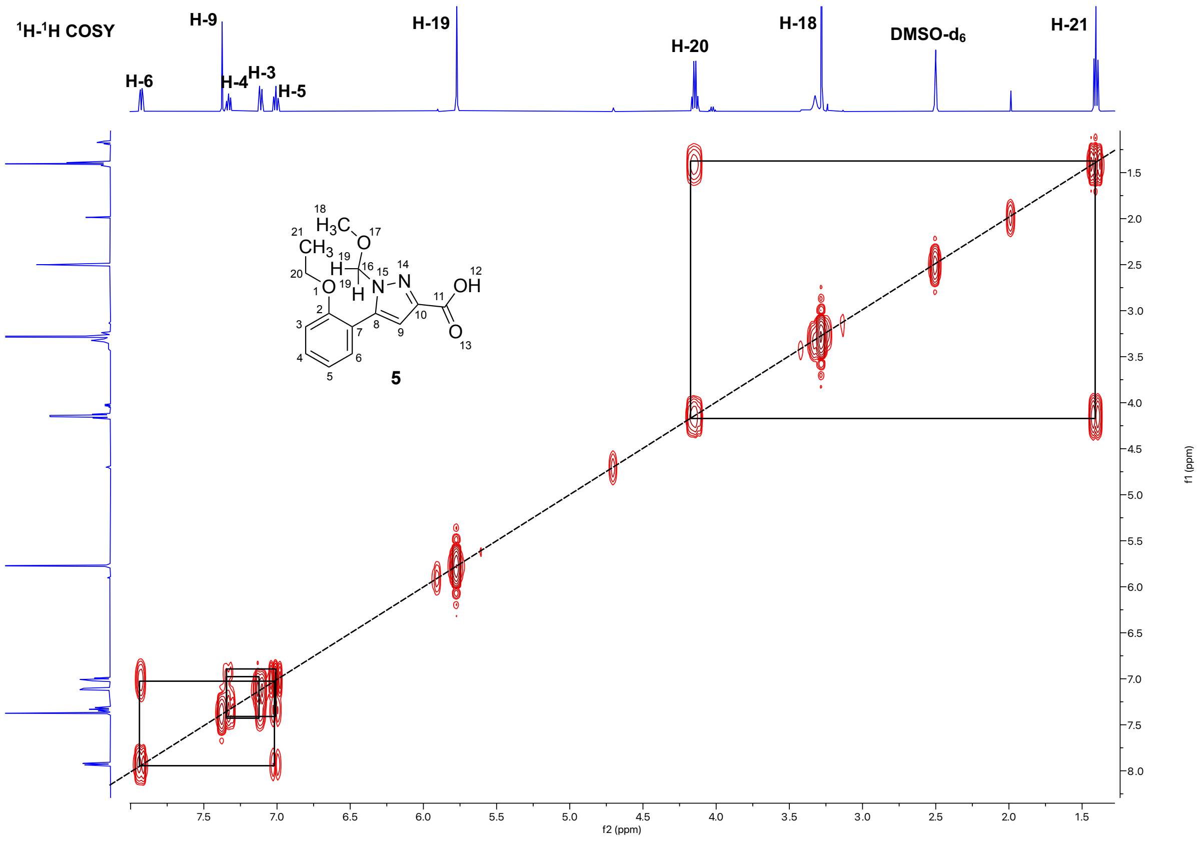

Figure S21: ^{1}H-^{13}C HSQC NMR (DMSO-d_6) for **5**

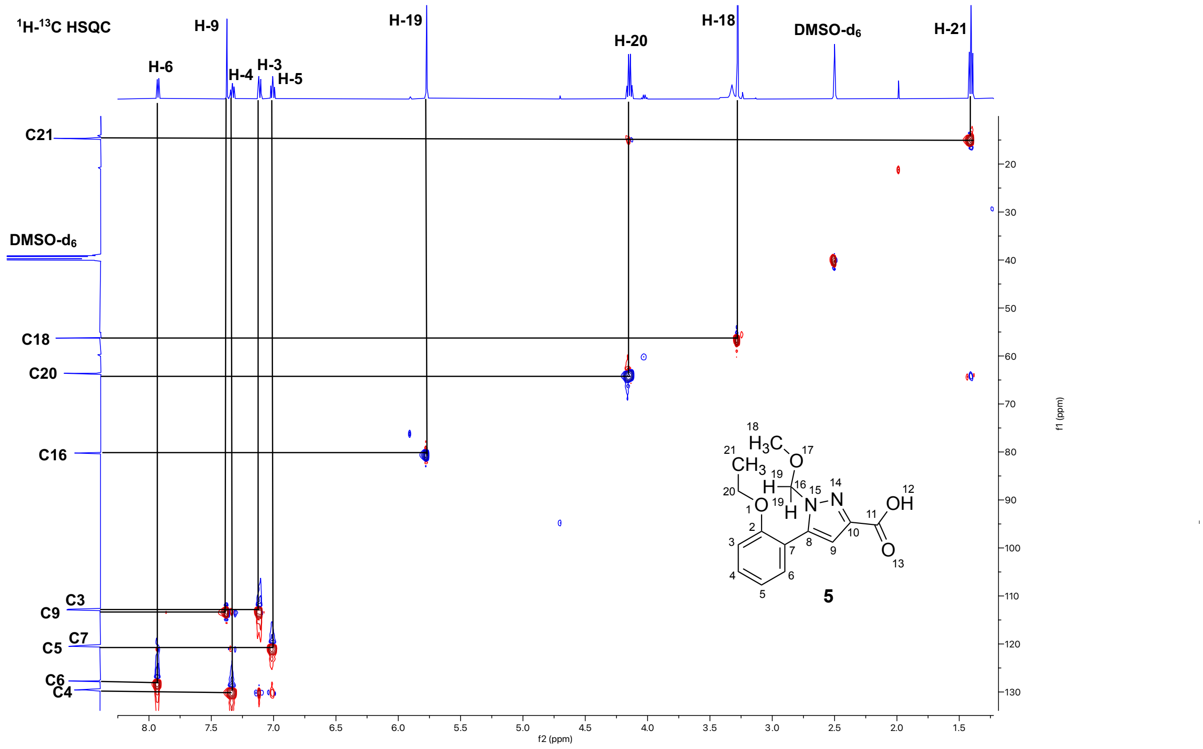

Figure S22: ^{1}H-^{13}C HMBC NMR (DMSO-d_6) of expanded region for **5**

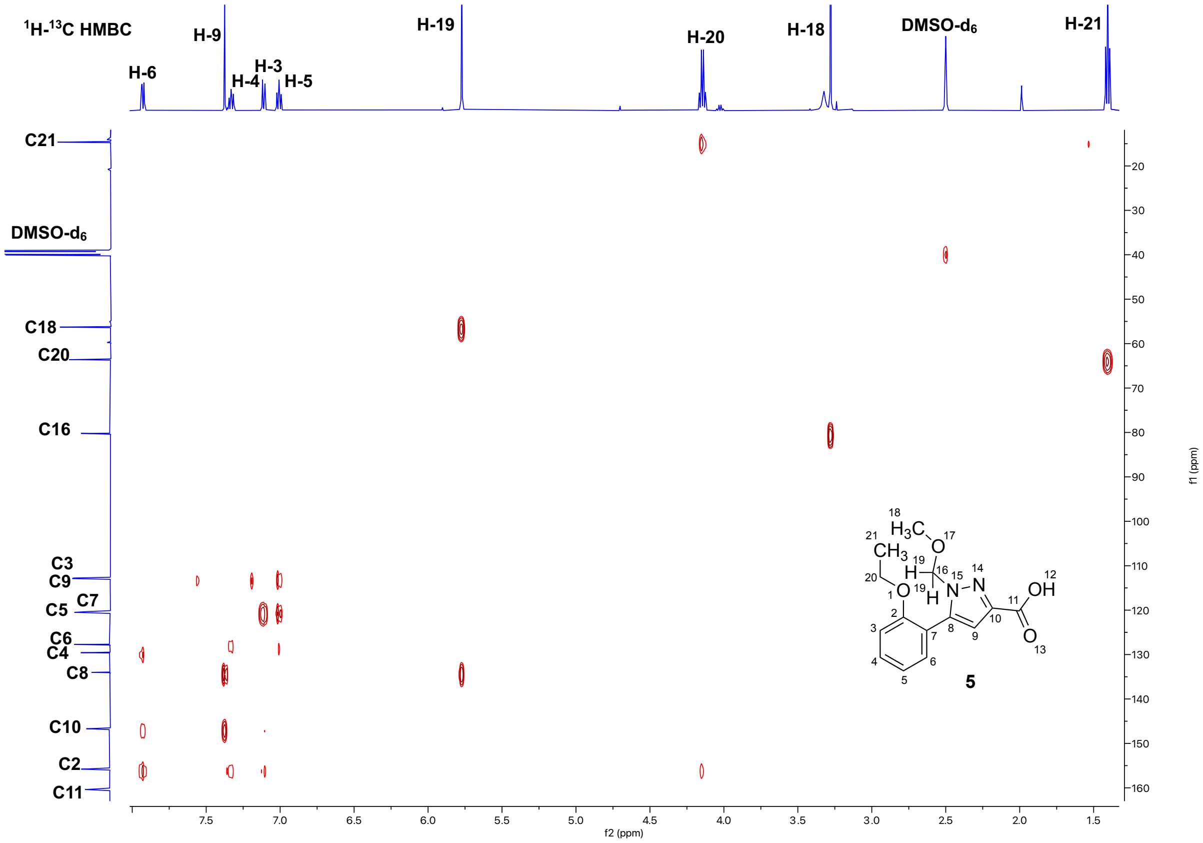

Figure S23: ^{1}H-^{13}C HMBC NMR (DMSO-d_6) of expanded region for **5**

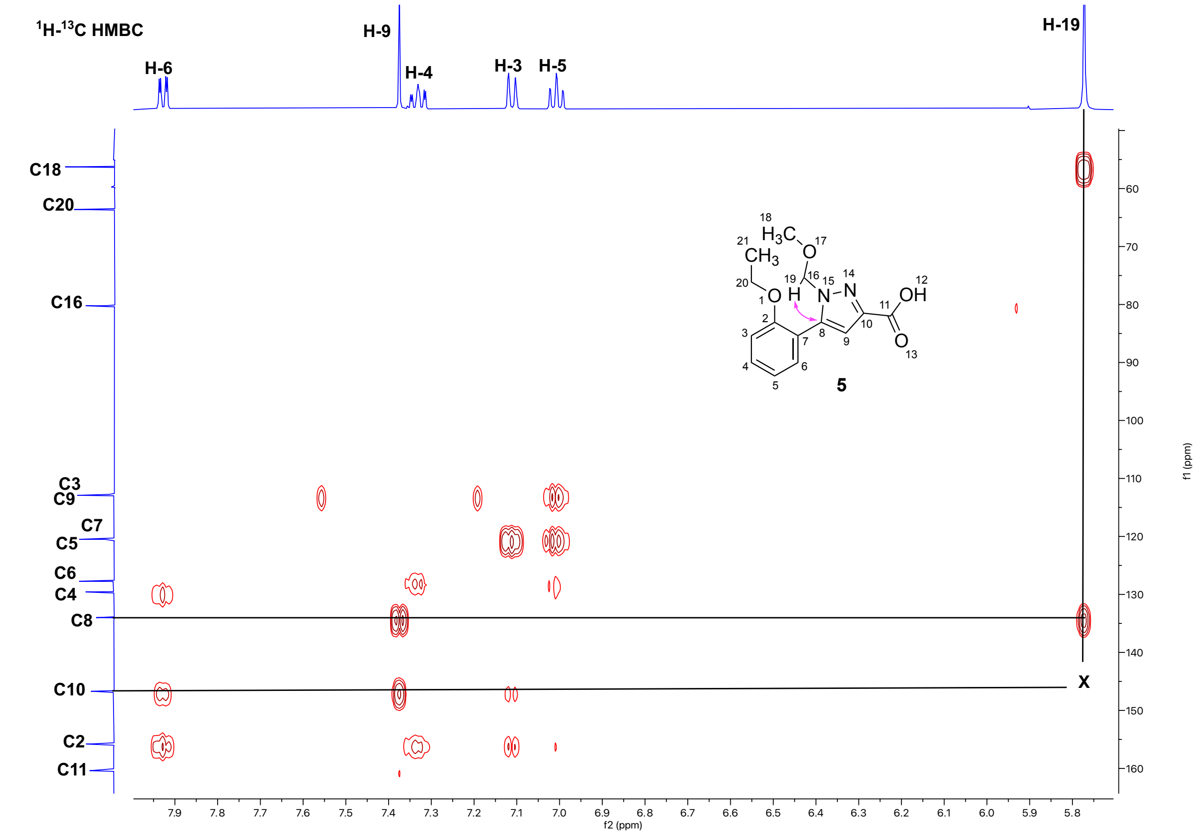

Figure S24: ^{1}H NMR (500 MHz, DMSO-d_6) for **6**

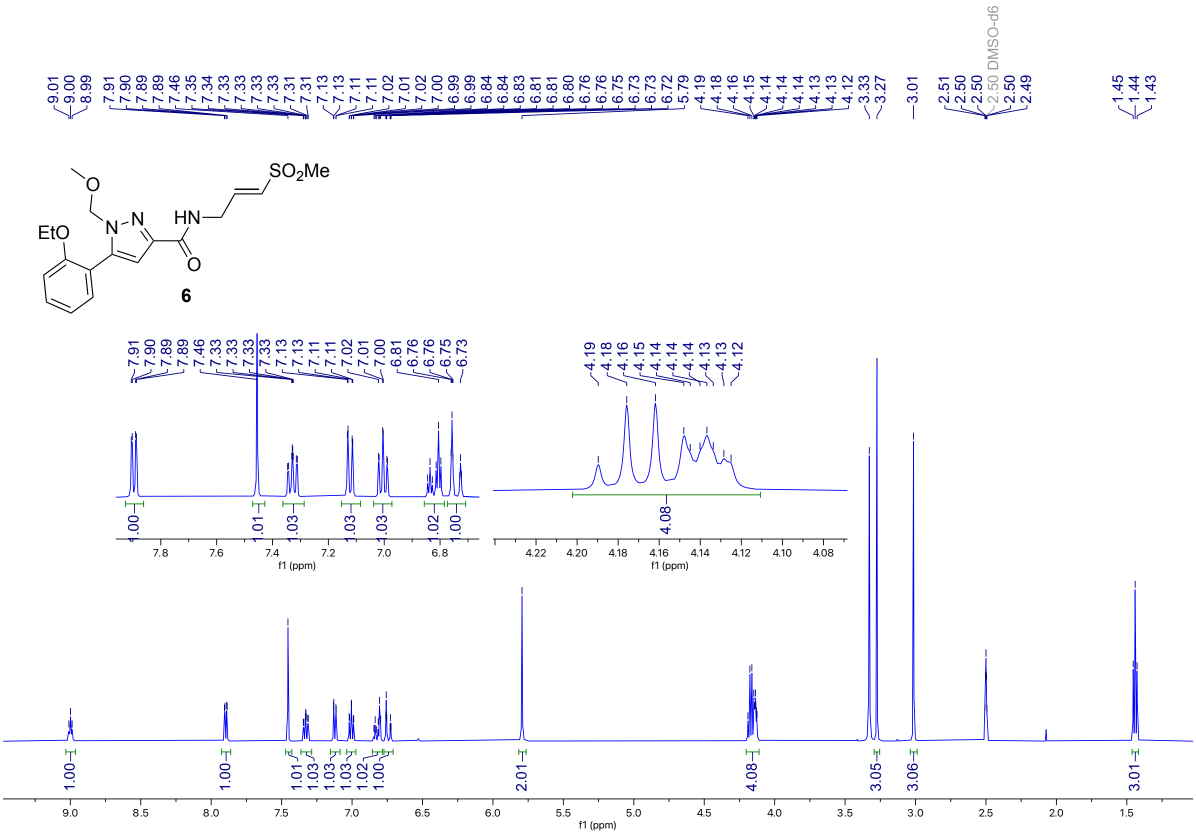

Figure S25: ^{13}C NMR (126 MHz, DMSO-d_6) for **6**

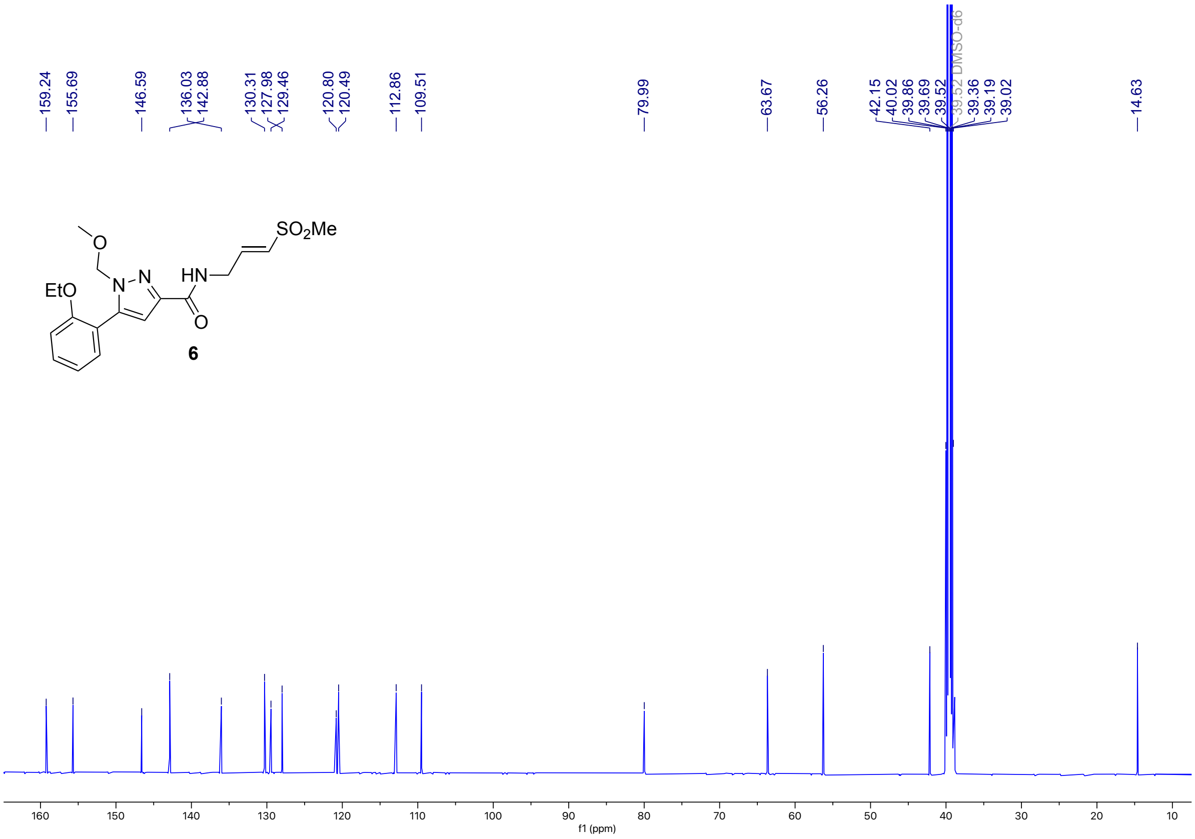

Figure S26: ^{13}C NMR (126 MHz, DMSO-d_6) for **7•TFA**

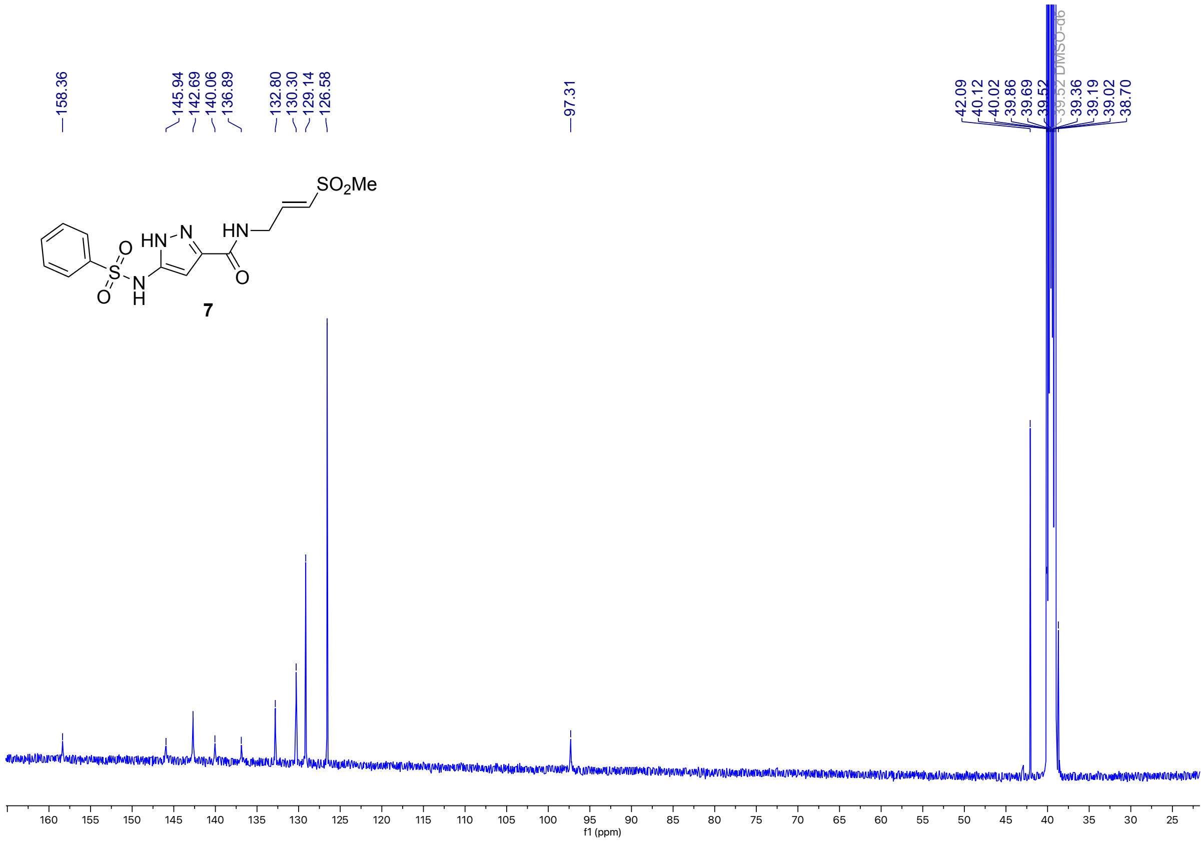

Figure S27: ^{1}H NMR (500 MHz, DMSO-d_6) for **8•TFA**

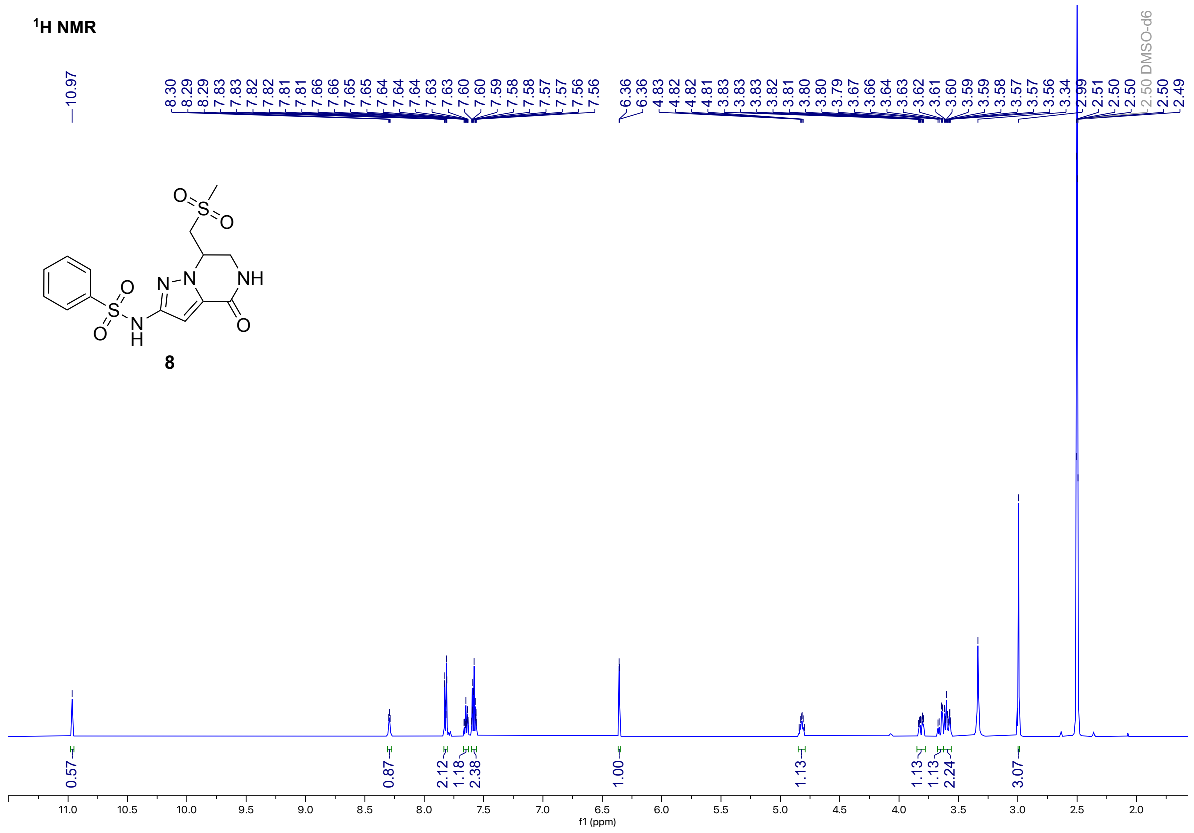

Figure S28: ^{13}C NMR (176 MHz, DMSO-d_6) for **8•TFA**

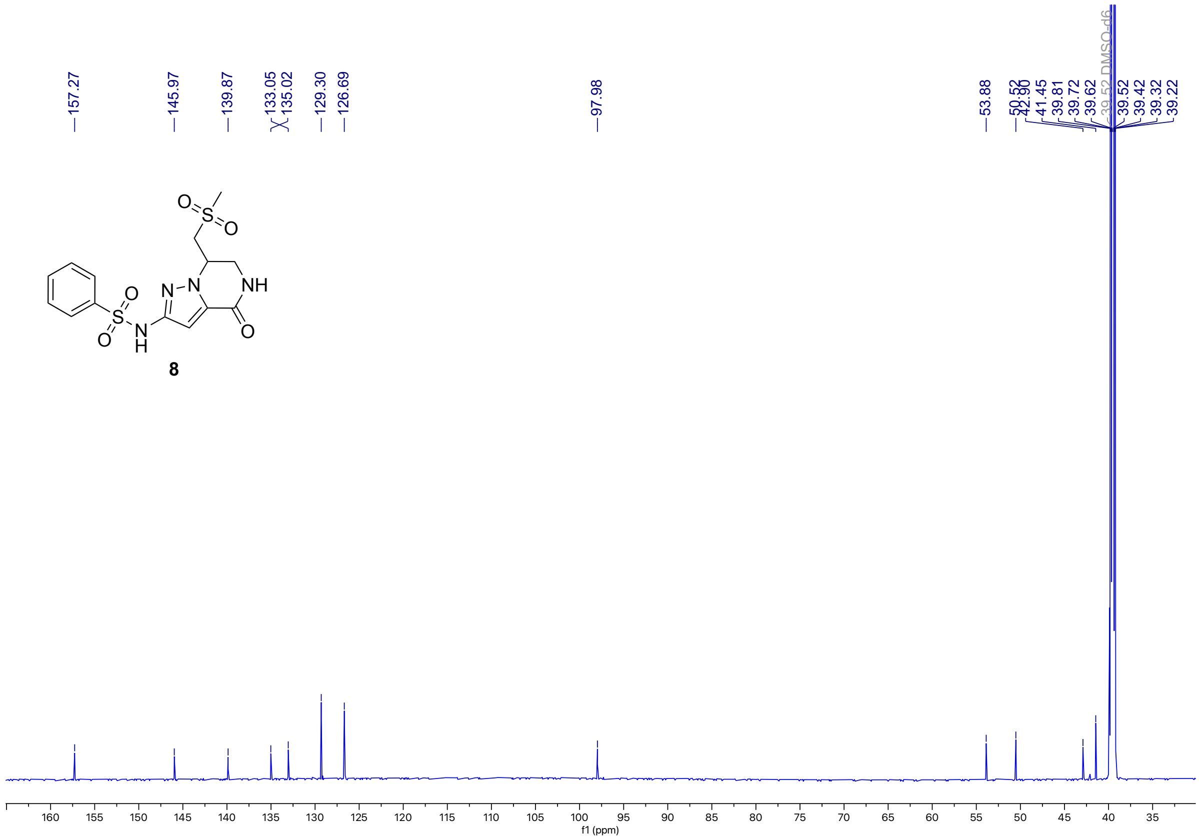

Figure S29: LC/MS purity for **1•TFA**

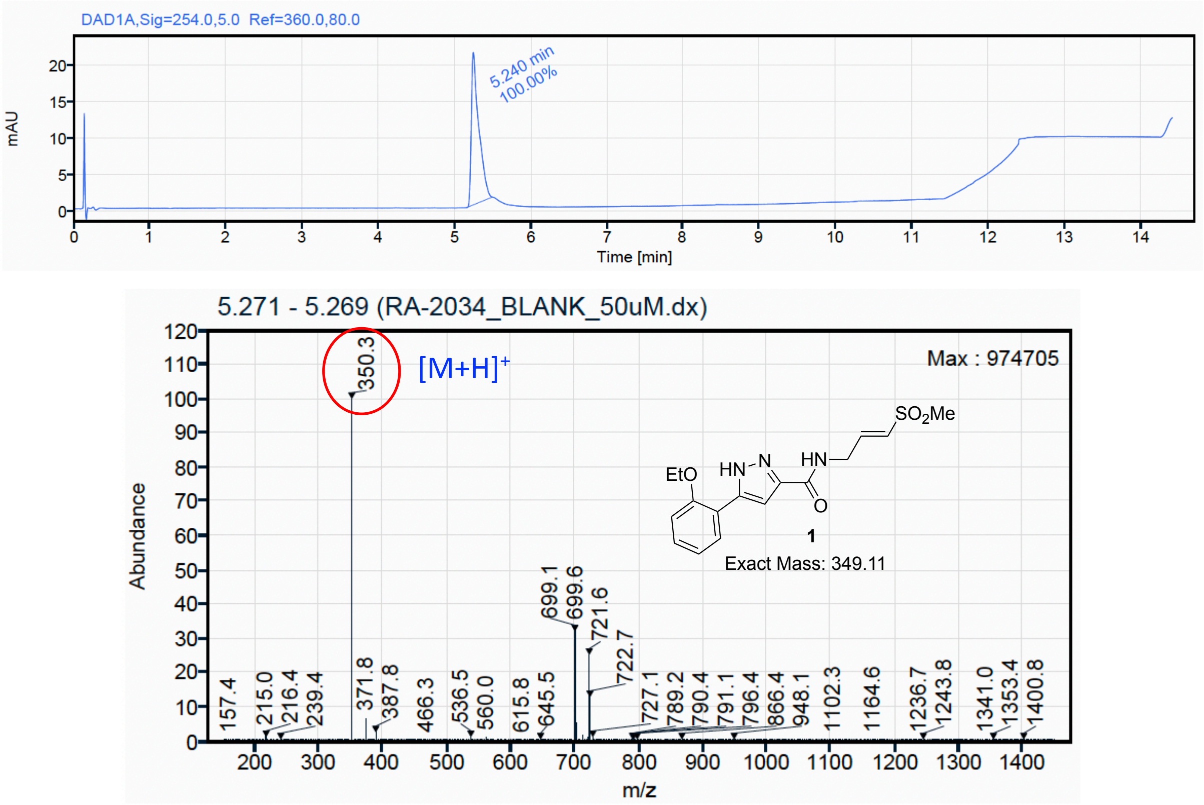

Figure S30: LC/MS purity for **2**

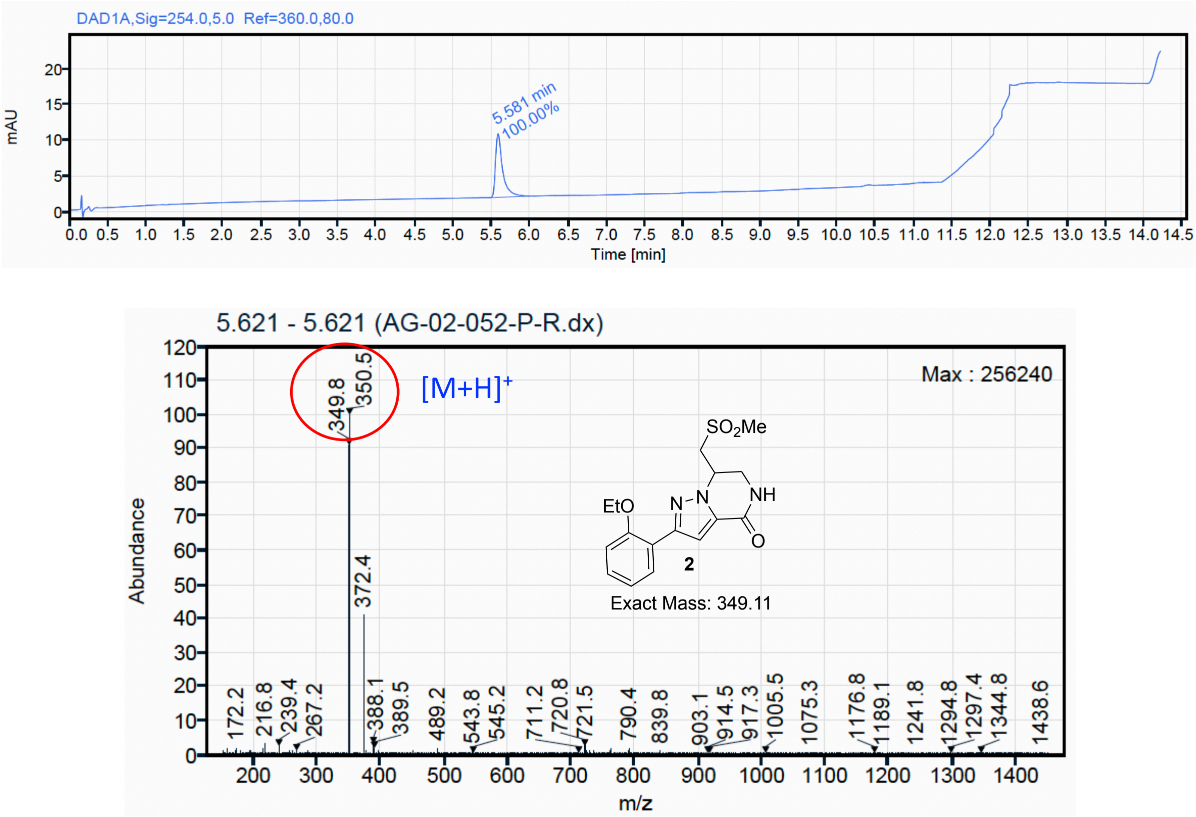

Figure S31: LC/MS purity for **7•TFA**

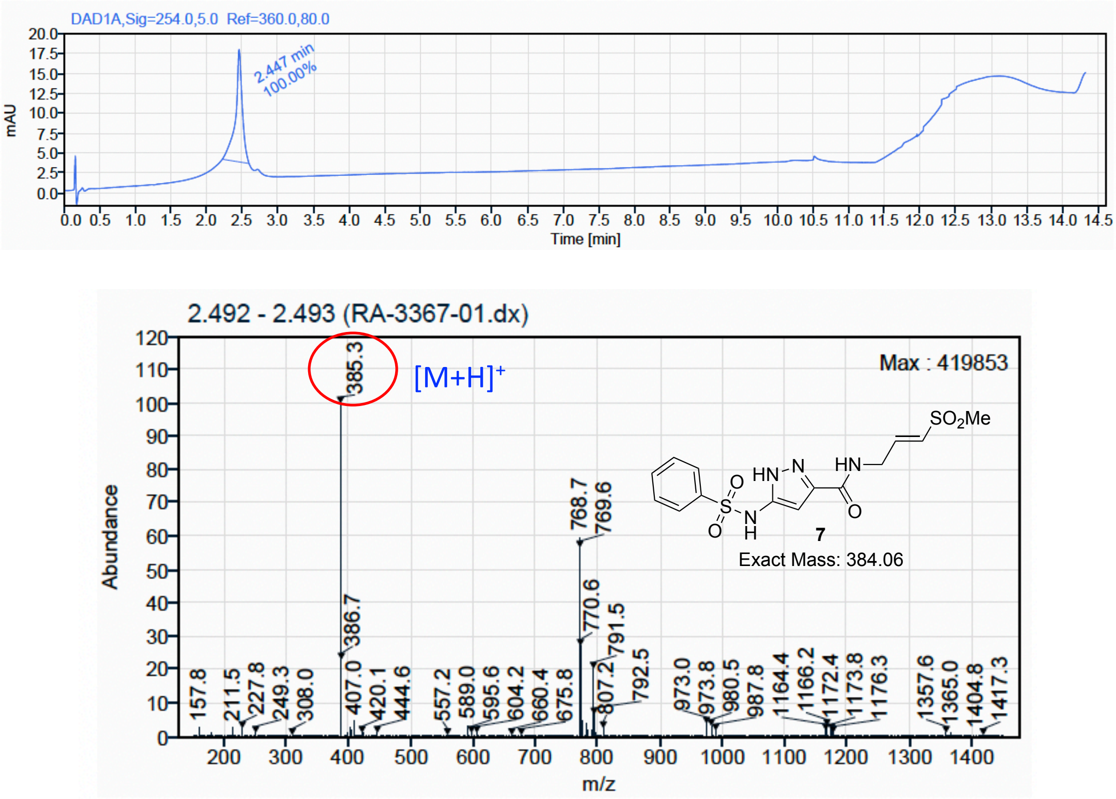

Figure S32: LC/MS purity for **8•TFA**

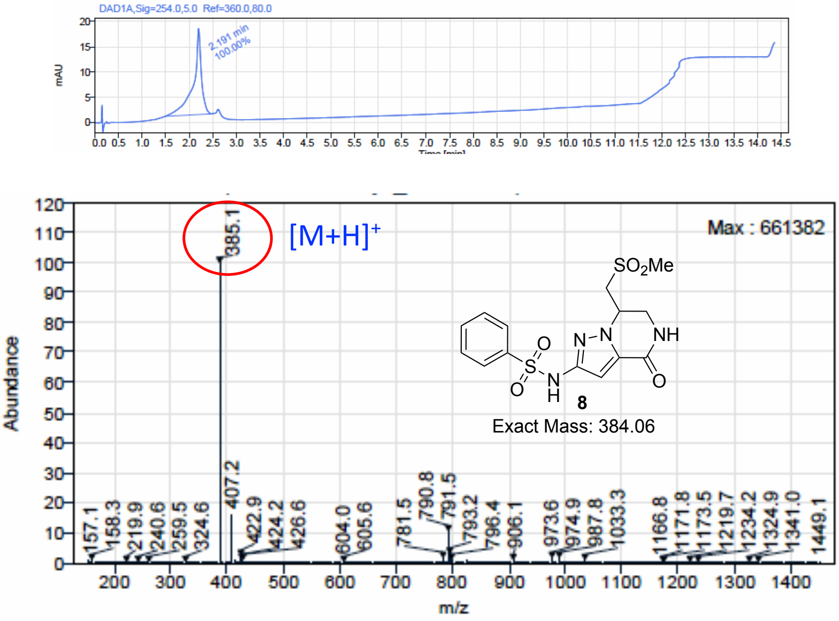

Figure S33: HRMS spectrum for **1•TFA**

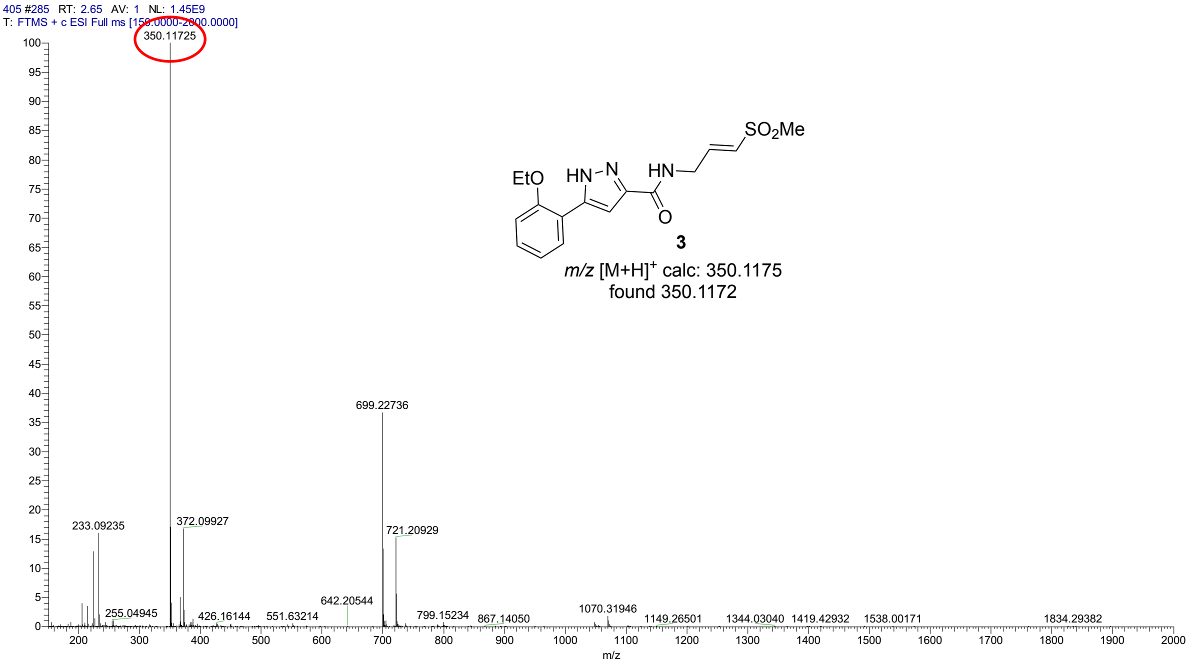

Figure S34: HRMS spectrum for **2**

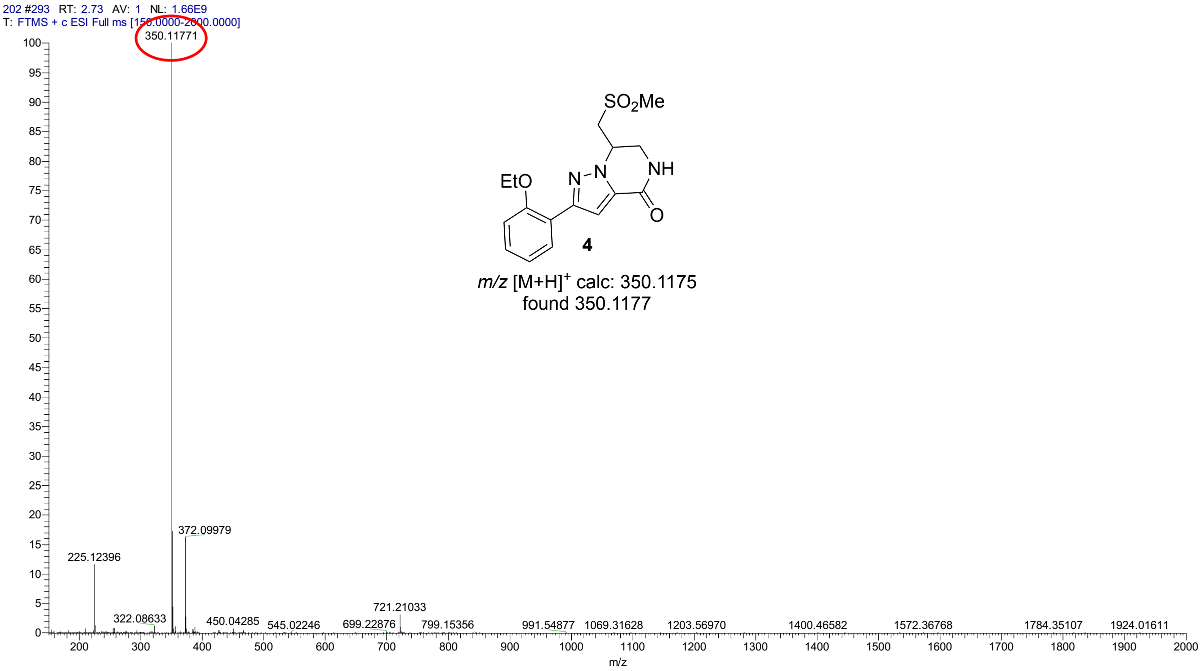

Figure S35: HRMS spectrum for **7•TFA**

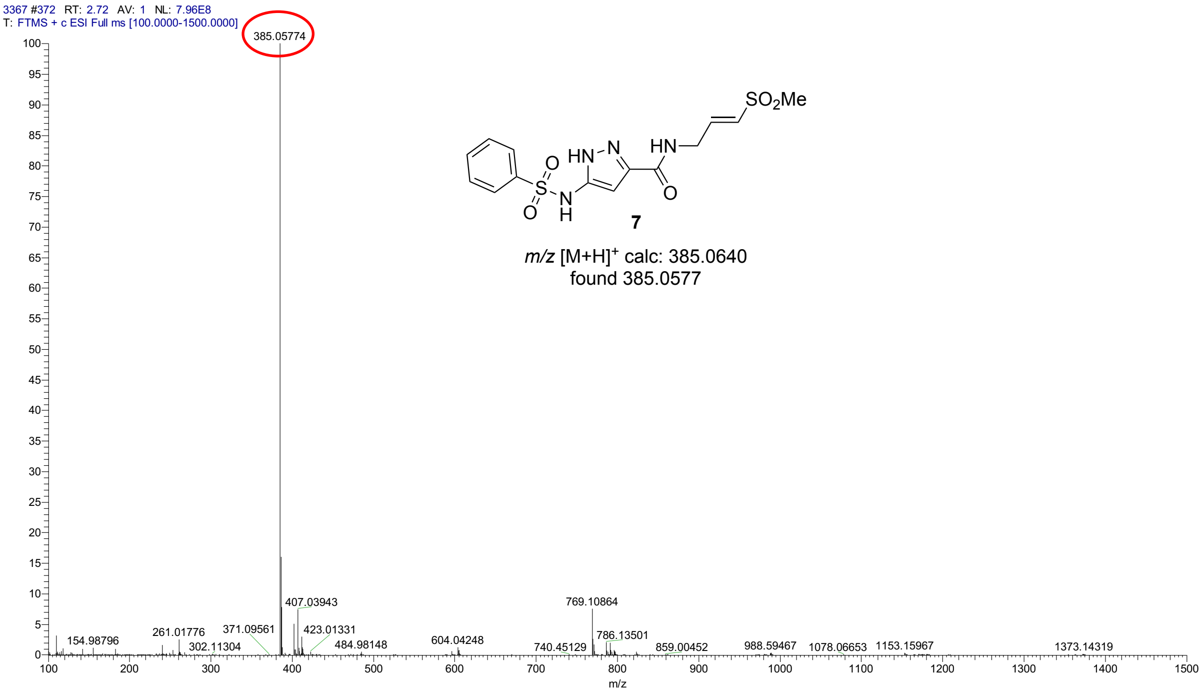

Figure S36: HRMS spectrum for **8•TFA**

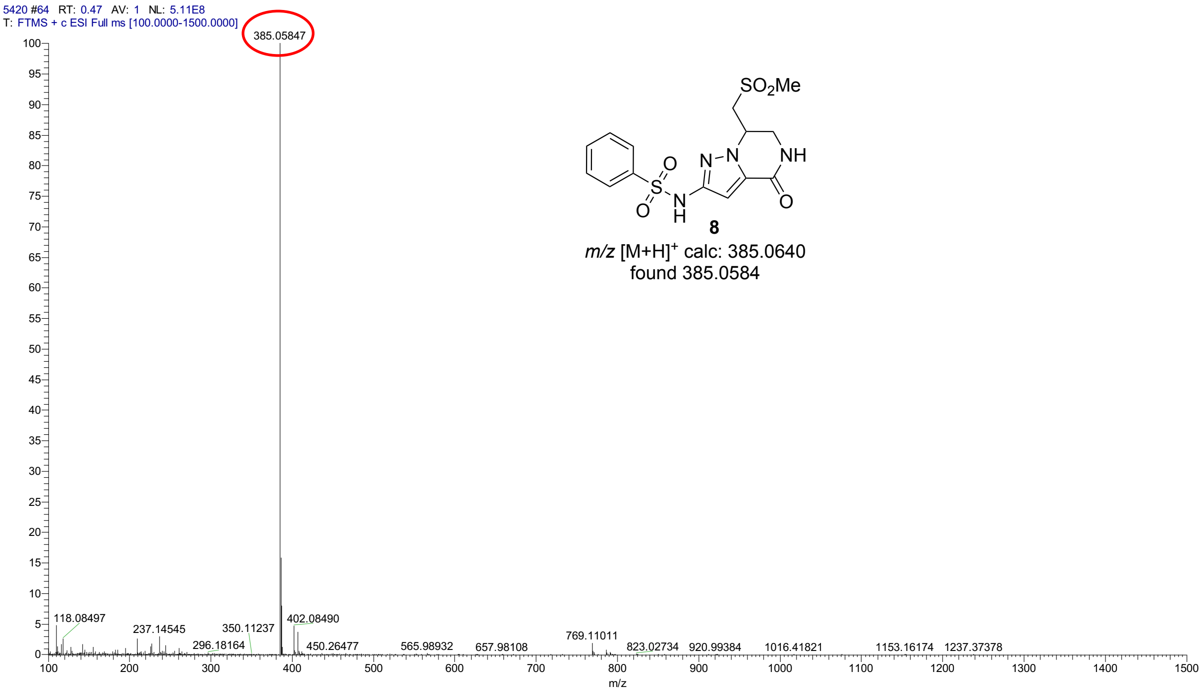

Supplement: Supplementary file 1 [file pharmaceuticals-17-00836-s001.zip › pharmaceuticals-3076068-supplementary.pdf]
